# RB1 and TP53 co-mutations correlate strongly with genomic biomarkers of response to immunity checkpoint inhibitors in urothelial bladder cancer

**DOI:** 10.1186/s12885-021-08078-y

**Published:** 2021-04-20

**Authors:** Ramon Gonzalez Manzano, Ana Catalan-Latorre, Antonio Brugarolas

**Affiliations:** 1 Molecular Genetics and Genomics Laboratory, Unidad de Consejo Genetico, Plataforma de Oncologia, Hospital Quironsalud Torrevieja, Pda. La Loma s/n, 03184 Torrevieja (Alicante), Spain; 2 Unidad de Farmacocinetica y Farmacoterapia Personalizada, Plataforma de Oncologia, Hospital Quironsalud Torrevieja, Pda. La Loma s/n, 03184 Torrevieja (Alicante), Spain; 3 Medical Oncology Department, Plataforma de Oncologia, Hospital Quironsalud Torrevieja, Pda. La Loma s/n, 03184 Torrevieja (Alicante), Spain

**Keywords:** RB1, TP53, Co-mutation, Immunity checkpoint inhibitor, Urothelial, Bladder, Cancer, Signature

## Abstract

**Background:**

Muscle invasive urothelial bladder carcinoma (MIBC) present RB1 and TP53 somatic alterations in a variable percentage of tumors throughout all molecular subtypes. MIBCs with neuroendocrine features have a high response rate to immunity checkpoint inhibitors (ICIs). Whether the presence of somatic co-alterations in these 2 genes in MIBCs is relevant to their responsiveness to ICIs is not known.

**Methods:**

The potential correlation of different genomic biomarkers of response to ICIs like tumor mutational burden (TMB), single nucleotide variants (SNV) predicted neoantigens, DNA damage response (DDR) genes, DNA somatic signatures and TILs infiltrate was explored in patients with somatic co-alterations in RB1 and TP53 (RB1&TP53) as compared with patients with no alterations in any (double wild type, DWT) or with alterations in just one of the 2 genes. The Cancer Genome Atlas (TCGA) pancancer BLCA dataset of cystectomy specimens (*n* = 407) with mutation, copy number alterations and transcriptomic (RNA sequencing) data as well as the IMVigor 210 study (*n* = 348) of metastatic urothelial bladder cancers treated with atezolizumab (PD-L1 inhibitor) with clinical response data containing transcriptomic (RNA sequencing), along with a subset (*n* = 274) with mutation and copy number data were used for this purpose. A novel tumor microenvironment metascore (TMM) was developed based in a LASSO regularized Cox model with predictive and prognostic ability.

**Results:**

Samples with co-altered RB1&TP53: a) were enriched in immunity effectors (CD8 cytotoxic lymphocytes, NK cells) and display higher scores of a T cell inflamed signature; b) have a higher TMB, higher number of SNV predicted neoantigens and higher TILs fractions; c) have a higher number of DDR mutated and deep deleted DDR genes; d) have DNA somatic signatures 2 and 13 related to APOBEC mutagenesis. Using the IMVigor 210 dataset, RB1&TP53 samples had the highest response rate to atezolizumab and a strong correlation with TMB and TMM. The consensus molecular subtype classification in the IMVigor 210 dataset showed a significant correlation with both the response to treatment (*p* = 0.001, Chisquare) and the presence of RB1 and TP53 genomic alterations (*p* < 0.001, Chisquare).

**Conclusions:**

RB1&TP53 co-alterations are strongly associated with genomic biomarkers of response to ICIs in MIBCs.

**Supplementary Information:**

The online version contains supplementary material available at 10.1186/s12885-021-08078-y.

## Background

Urothelial bladder carcinoma is one of the most commonly diagnosed cancers in the US and Europe. Approximately 25% of urothelial bladder cancers are muscle invasive (MIBC) or metastatic [1]. The treatment of locally advanced MIBC is platinum based chemotherapy followed by radical cystectomy with bilateral pelvic lymph node dissection. However, for patients not eligible or refractory to cisplatin, treatment with immunity checkpoint inhibitors (ICIs) can induce good and durable responses both in the neoadjuvant and metastatic settings [2–5]. MIBCs have been extensively characterized from a genomic perspective. They are heterogeneous tumors and have a high somatic mutation rate [1, 6]. Transcriptome profiling has unveiled different molecular subtypes which have been the subject of several molecular classifications [7–9]. Genomic studies have also pinpointed the enrichment of specific genomic alterations in the different molecular subtypes, supporting the view that the molecular subtypes are different disease entities [10]. Recently, a multidisciplinary team of experts has made available a molecular subtypes consensus classification that has taken into account six previously published classification systems [11]. This effort is of great interest for clinicians and researchers alike and can facilitate its clinical use regarding the prognosis and therapy of patients with MIBC.

A small subset of patients with advanced MIBC has been identified with a high survival probability after treatment with atezolizumab [12]. Specifically patients with neuroendocrine markers are among the best responders with an overall 72% response rate [12]. The presence of TP53 and RB1 somatic alterations is a known hallmark of poorly differentiated neuroendocrine carcinomas [13]. Whether the presence of mutations or other genomic alterations in these genes can contribute to the good response of these tumors to ICIs is not known. There is preliminary evidence suggesting that molecular aberrations of both TP53 and RB1 may impact the tumor microenvironment as shown in a global study of more than 10,000 patients from The Cancer Genome Atlas (TCGA) that also included patients with MIBC [14]. However, a selective analysis of the potential influence of the different types of somatic alterations in TP53 and RB1 (either alone or in combination) in the tumor microenvironment of MIBC, and the potential correlation of the co-alteration of these 2 genes with genomic predictors of response to ICIs has not been performed and it could be of help to understand the good therapeutic response to ICIs in tumors with neuroendocrine markers. Further, not only MIBC patients with neuroendocrine markers have mutations in TP53 (almost 100%) and RB1 (> 60%). Other molecular subtypes of MIBC according to the latest consensus classification also have mutations in these 2 genes: Luminal papillary (TP53 32%, RB1 5%), luminal nonspecified (TP53 45%, RB1 5%), luminal unstable (TP53 76%, RB1 22%), stroma-rich (TP53 28%, RB1 21%) and basal/squamous (TP53 61%, RB1 25%) [11]. For this reason a better understanding of the molecular interactions of these genes with the tumor microenvironment and with other genomic predictors of response is of potential clinical relevance as it may contribute to explain at least in part the sensitivity to immunotherapeutic agents, not just in MIBCs with neuroendocrine markers but also in the full spectrum of MIBCs, as all molecular subtypes have different proportions of co-alterations in TP53 and RB1. In this study we tackle this objective by reanalyzing the TCGA data focusing in the somatic alterations of these 2 genes, using mutation, copy number and transcriptome data and complementing the study with data from the IMVIGOR 210 study, containing clinical response along with mutations, copy number and transcriptomic data to verify relevant findings [15].

## Results

### Modulation of the cell populations in the tumor microenvironment by genomic alterations in RB1 and TP53

We studied the urothelial bladder cancer dataset from TCGA containing 407 patients with mutational, copy number and transcriptomic data. Taking into account mutations and copy number alterations: 187 patients (45.9%) were wild type for both TP53 and RB1 (from now on double WT or DWT); 30 patients (7.4%) had RB1 genomic alterations only (with no TP53 molecular aberrations); 121 (29.7%) had TP53 genomic alterations only (with no RB1 alterations) and 69 (17%) had genomic alterations concurrently in both genes: RB1 and TP53.

The signature scores of the 10 cell populations and the 2 immunological signatures (Ayers, also called the T cell inflamed signature, and the cytolysis signature) studied were first obtained from the normalized transcriptomic data from TCGA as explained in the Methods section. These scores were compared in the MIBC patients according to the TP53 and RB1 mutational status using the Wilcoxon rank sum test (two-sided). Patients with genomic alterations in RB1 only did not show significant differences in any of the 12 scores tested (FDR > 0.05 for all comparisons) when compared with the DWT scores. Samples with TP53 alterations only showed significant differences (lower values) in the endothelial cells score (FDR = 0.004) as compared with DWT. Interestingly, patients with concurrent alterations in RB1 and TP53 showed the largest differences in 3 cell populations: significantly higher scores in cytotoxic lymphocytes (FDR = 0.00495) and NK cells (FDR = 0.0081), and significantly lower scores in endothelial cells (FDR = 0.0013), all 3 cell populations as compared with DWT patients (WT for both RB1 and TP53). In addition, the T cell inflamed signature had a significantly higher score in these patients with alterations in both RB1 and TP53 as compared with DWT (FDR = 0.02).

To gain further insight into the differences observed in the cell populations present in the tumor microenvironment according to the mutational status of TP53 and RB1, we made additional comparisons. Firstly, all RB1 deleterious mutations without taking into account RB1 homozygous deletions (63 patients, 15.5%) against RB1 WT, with or without TP53 mutations (308 patients, 75.7%). Significantly higher scores were found for the following cell populations in the RB1 mutated samples: cytotoxic lymphocytes (FDR = 0.00036), NK cells (FDR = 0.0005), B cell lineage (FDR = 0.001), monocytic lineage (FDR = 0.0047); and also significantly higher scores for the 2 immunological signatures: T cell inflamed signature (FDR = 0.0005) and the cytolysis signature (FDR = 0.00076). As expected from the previous results, the proportion of TP53 mutated patients was higher among the RB1 deleterious mutated samples than among the RB1 WT (OR 4.80, 95% CI 2.53–9.51, *p* = 3.219E-07, Fisher’s exact test). Then, we considered the RB1 homozygous deletions (HD) (36 patients, 8.8%) and comparing with the RB1 WT samples as before, no such differences were found in the aforementioned cell populations or the immunological signatures (FDR > 0.05 for all comparisons) in spite of also having a higher proportion of TP53 mutated patients in the RB1 HD samples as compared with the RB1 WT (OR 2.57, 95% CI 1.2–5.67, *p* = 0.011, Fisher’s exact test). Furthermore, the proportion of TP53 mutated samples is not significantly different between RB1 deleterious mutations and the RB1 HD samples (OR 1.86, 95% CI 0.70–4.91, *p* = 0.18, Fisher’s exact test). The only cell population that had significantly different scores between the RB1 HD samples and the RB1 WT was the endothelial cells (FDR = 0.041), with lower scores in RB1 HD than in RB1 WT. Secondly, we also compared the TP53 truncating mutations (including nonsense, splicing and frameshift mutations, 67 patients, 16.5%) against the TP53 WT (217 patients, 53.3%). Three cell populations had significantly higher scores: cytotoxic lymphocytes (FDR = 0.0066), NK cells (FDR = 0.01) and monocytic lineage (FDR = 0.0066), and also significantly higher scores were observed for the 2 immunological signatures (T cell inflamed FDR = 0.0066, and cytolysis FDR = 0.041). Interestingly when we made the same comparison but using the TP53 missense mutants (116 patients, 28.5%) against the TP53 WT, none of the previous statistically significant differences among the 3 cell populations and the 2 immunological signatures were observed (FDR > 0.05 for all comparisons), and only a significantly lower score was noted for the endothelial cell population (FDR = 8.3E-05). Whereas the overall proportion of all RB1 mutants + RB1 HD was higher in both the TP53 truncated mutants and the TP53 missense mutants with respect to the TP53 WT samples (*p* < 0.05 for the 2 comparisons, Fisher’s exact test), we wondered whether the proportion of RB1 HD was higher between the TP53 missense and the TP53 WT samples. Indeed, a higher proportion of RB1 HD was present in the TP53 missense mutants as compared with the TP53 WT (OR 3.32, 95% CI 1.43–7.81, *p* = 0.0025, Fisher’s exact test). However, no significant differences were seen between the proportion of RB1 HD in the TP53 truncating mutations and that of TP53 WT (OR 1.60, 95% CI 0.48–4.71, *p* = 0.397, Fisher’s exact test), providing a potential explanation for the lower impact of TP53 missense mutants in the cell populations of the tumor microenvironment.

In summary, the increase in cytotoxic lymphocytes and NK cells as well as the higher T cell inflamed signature scores were the cell populations and the signature, respectively, that showed the most consistent variation in the tumor microenvironment of samples affected by mutations in both genes, RB1 and TP53. In addition, RB1 HD seem to have a lower impact in the cell populations in the tumor microenvironment, affecting only the endothelial cell population (lower scores).

### Signaling pathways involved in concurrent genomic alterations in RB1 and TP53

Next, we characterized the signaling pathways affecting samples with genomic alterations in RB1 and TP53 simultaneously, as these showed a significant increase in cytotoxic lymphocytes and NK cells, probably the most interesting and powerful effectors of the immunological response, and higher T cell inflamed signature scores, which have been related to interferon gamma responsive genes and to response to ICIs [16, 17].

We generated from the normalized transcriptome data, as explained in the Methods section, the single sample GSEA (ssGSEA) scores of the hallmark geneset collection of 50 signatures from the Molecular Signatures Database v5.1 (Broad Institute) for the 407 TCGA MIBC patients under study.

A number of hallmark pathways were found significantly up or downregulated when comparing the RB1 and TP53 concurrently altered samples with the DWT tumors at a FDR < 0.10 (Table 1).

**Table 1:** Hallmark pathways differentially expressed ssGSEA scores (FDR < 0.10) between concurrent RB1 and TP53 genomically altered and DWT (Wilcoxon rank sum test, two-sided)

Hallmark pathways	Regulation	FDR
E2F_TARGETS	UP	2.85E-16
G2M_CHECKPOINT	UP	2.00E-14
MITOTIC_SPINDLE	UP	2.52E-13
SPERMATOGENESIS	UP	3.65E-10
NOTCH_SIGNALING	DOWN	1.31E-09
MYC_TARGETS_V1	UP	1.87E-09
ADIPOGENESIS	DOWN	7.90E-07
PANCREAS_BETA_CELLS	UP	1.27E-06
UV_RESPONSE_UP	UP	1.26E-05
DNA_REPAIR	UP	2.85E-05
P53_PATHWAY	DOWN	4.67E-05
UNFOLDED_PROTEIN_RESPONSE	UP	4.77E-05
MTORC1_SIGNALING	UP	0.00025
XENOBIOTIC_METABOLISM	DOWN	0.00037
PEROXISOME	DOWN	0.00085
FATTY_ACID_METABOLISM	DOWN	0.00222
BILE_ACID_METABOLISM	DOWN	0.023
ANDROGEN_RESPONSE	DOWN	0.028
OXIDATIVE_PHOSPHORYLATION	DOWN	0.028
REACTIVE_OXIGEN_SPECIES_PATHWAY	DOWN	0.032
MYC_TARGETS_V2	UP	0.034
GLYCOLYSIS	UP	0.051
ALLOGRAFT_REJECTION	UP	0.058
INTERFERON_GAMMA_RESPONSE	UP	0.060
TGF_BETA_SIGNALING	DOWN	0.065
MYOGENESIS	DOWN	0.075
INTERFERON_ALPHA_RESPONSE	UP	0.075
INFLAMMATORY_RESPONSE	UP	0.078

A highly significant upregulation of pathways related to cell proliferation and cell division was clearly apparent (Mitotic spindle, G2M checkpoint, E2F targets and MYC targets) and expected in the RB1 and TP53 altered samples. Of note, MYC as a master transcription factor regulating metabolic reprogramming seems to be related to a significant increase in glycolysis and a decrease in oxidative phosphorylation (the Warburg effect).

A number of significantly altered pathways is related to a favorable immune response in patients treated with ICIs (upregulation of interferon gamma [15–17], interferon alpha [18], allograft rejection, inflammatory response and downregulation of TGFB signaling [15]). And this observation correlates well with the increase in immunological effectors (cytotoxic lymphocytes and NK cells) in RB1 and TP53 concurrently altered MIBC samples (see previous Results section).

DNA repair and UV_REPONSE_UP are also significantly overrepresented in the RB1 and TP53 co-altered samples as well as the MTORC1_SIGNALING. It is also worth noticing that the signature PANCREAS_BETA_CELLS is also upregulated likely capturing some of the neuroendocrine features present in the neuroendocrine-like molecular subtype.

As in the previous analysis on the cellular populations in the tumor microenvironment we found that when comparing samples with TP53 missense mutations with TP53 WT, the results did not show a significant increase in the 3 cell populations (cytotoxic lymphocytes, NK cells and monocytic lineage) and the 2 signatures (T cell inflamed and cytolysis) as was shown with the comparison of TP53 truncating mutations against the TP53 WT samples, we wondered whether there was a functional difference in the TP53 truncated set of mutants (67 patients) as compared with the TP53 missense mutants (116 patients). Thus, we compared the 50 ssGSEA scores of the cancer hallmark genesets between the 2 sets of mutants (truncating and missense) in order to identify potential functional differences in these pathways. However, we did not find any significant difference in any of the 50 comparisons between the 2 sets of TP53 mutants (FDR > 0.10 for all comparisons). Hence, as previously noted, the observed differences in the 3 cell populations and the 2 signatures of these 2 sets of mutants with the TP53 WT samples are likely to be attributable to the higher proportion of RB1 HD in the TP53 missense mutants.

### Tumor mutational burden (TMB) and TIL fractions in samples with RB1 and TP53 genomic alterations

As a higher TMB has been associated with a better response with ICIs, next we explored whether the TMB as measured by the non-silent mutation rate (mutations per Mb) and as a consequence, the single nucleotide variant (SNV) predicted neoantigens were increased, particularly in the MIBC tumors with a concurrent RB1 and TP53 genomic alteration. We also explored whether TIL fractions were significantly different in this tumor populations.

The results are shown in Table 2.

**Table 2:** Means and standard deviations of SNV predicted neoantigens, mutations/Mb of non-silent mutations and TIL fractions in the indicated tumor populations

	SNVneoantigens (predicted)a	NonsilentMUTS (muts/Mb)b	TILs fraction (%)c
**DWT**	84.7 ± 86.3	5.0 ± 5.3	6.0 ± 6.4
**RB1 only**	141.6 ± 145.1	8.2 ± 7.8	7.8 ± 6.6
**TP53 only**	102.7 ± 81.4	6.4 ± 4.7	7.6 ± 6.3
**RB1&TP53**	135.6 ± 101.4	8.4 ± 6.2	9.1 ± 7.8
Kruskal-Wallis (*p* value)	a < 0.001	b < 0.001	c 0.002
**RB1 WT**	91.2 ± 84.8	5.5 ± 5.1	6.6 ± 6.4
**RB1 MUT**	117.1 ± 85.4	7.3 ± 5.2	8.6 ± 8.2
**RB1 HD**	180.3 ± 158.5	10.5 ± 8.8	8.7 ± 5.6
Kruskal-Wallis (*p* value)	a < 0.001	b < 0.001	c 0.015
**TP53 WT**	93.5 ± 99.3	5.5 ± 5.9	6.3 ± 6.4
**TP53 TRUNCATED**	106.6 ± 90.0	7.1 ± 5.9	9.1 ± 8.3
**TP53 MISSENSE**	123.7 ± 91.9	7.5 ± 5.1	7.8 ± 6.1
Kruskal-Wallis (p value)	a < 0.001	b < 0.001	c 0.002

As shown in Table 2, the number of predicted SNV neoantigens and the TMB (Non-silent mutations/Mb) were significantly higher in RB1 only (*p* = 0.005 and *p* = 0.003 respectively, Wilcoxon rank sum test two sided) and in RB1&TP53 altered tumors (*p* < 0.001 and p < 0.001 respectively, Wilcoxon rank sum test two sided) as compared with DWT. TIL fractions were significantly higher in RB1&TP53 tumors as compared with DWT (*p* = 0.001, Wilcoxon rank sum test two sided).

RB1 mutants and RB1 HD had higher predicted SNV neoantigens and TMB than RB1 WT as well as a higher TILs fraction. Whereas the number of predicted neoantigens was significantly higher in RB1 HD than in RB1 mutant (*p* = 0.036, Wilcoxon rank sum test two sided), the TMB was higher (in RB1 HD) but not significantly different than in RB1 mutant (*p* = 0.053 Wilcoxon rank sum test two sided). The TILs fraction were not significantly different between RB1 HD and RB1 mutants (*p* = 0.525 Wilcoxon rank sum test two sided).

Regarding the TP53 mutants, TP53 truncated (SNV neoantigens *p* = 0.004, TMB *p* < 0.001, TILs fraction *p* = 0.003) and TP53 missense (SNV neoantigens *p* < 0.001, TMB *p* < 0.001, TILs fraction *p* = 0.01) were both significantly higher (for SNV neoantigens, TMB and TILs fraction) than the TP53 WT tumors (Wilcoxon rank sum test two sided). However, there were no significant differences in SNV neoantigens, TMB and TILs fraction between TP53 truncated and TP53 missense mutants (*p* > 0.4 for all comparisons, Wilcoxon rank sum test). Even though the TILs fraction is highest in TP53 truncated mutants, and this result is concordant with the increase in cytotoxic lymphocytes and NK cells in these samples as compared with the TP53 WT shown above.

In summary, the presence of a concurrently genomically altered RB1 and TP53 in MIBC is significantly associated with a higher SNV neoantigen load, a higher TMB and also a higher TILs fraction than tumors with DWT, making this feature of potential interest in the prediction of sensitivity to ICIs along with the previously described findings.

### Relationship of genomic alterations in other DNA damage response (DDR) genes with RB1 and TP53 genomic alterations

It has been previously reported in MIBC that the presence of deleteriously mutated DDR genes is associated with a better clinical response to ICIs [19]. Hence we decided to explore whether there was a higher incidence of genomic alterations in DDR genes in TP53 and RB1 concurrently mutated tumors.

The results are shown in Table 3.

**Table 3:** Mean number of deleteriously mutated and deleted DDR genes (± sd) in MIBC patients with RB1 and TP53 alterations

	No. Mutated DDR genes (a)	No. Deleted DDR genes (b)
**DWT**	2.2 ± 3.2	1.0 ± 2.0
**RB1 only**	3.2 ± 4.0	3.0 ± 3.7
**TP53 only**	3.2 ± 2.6	1.2 ± 2.3
**RB1 & TP53**	3.7 ± 2.8	2.2 ± 3.3
Kruskal-Wallis (*p* value)	a < 0.001	b < 0.001
**RB1 WT**	2.6 ± 3.0	1.1 ± 2.1
**RB1 MUT**	3.2 ± 2.8	1.9 ± 3.2
**RB1 HD**	4.1 ± 3.7	3.4 ± 3.6
Kruskal-Wallis (*p* value)	a 0.003	b < 0.001
**TP53 WT**	2.4 ± 3.3	1.3 ± 2.4
**TP53 TRUNCATED**	3.8 ± 2.6	1.4 ± 2.8
**TP53 MISSENSE**	3.3 ± 2.7	1.5 ± 2.4
Kruskal-Wallis (*p* value)	a < 0.001	b 0.47

The results of Table 3 consider that TP53 is a DDR gene (but not RB1). There is a significant increase in the number of both mutated and deep deleted DDR genes in the concurrently altered RB1 and TP53 as compared with DWT (*p* < 0.001 for both comparisons, Wilcoxon rank sum test). In addition, RB1 only samples (but not TP53 only, *p* = 0.452) have a significantly higher number of deep deleted DDR genes (*p* = 0.001, Wilcoxon rank sum test two sided). Conversely, TP53 only tumors (but not RB1 only, *p* = 0.155) have also a significantly higher number of mutated DDR genes (*p* < 0.001, Wilcoxon rank sum test two sided).

Considering MIBC tumors according to the RB1 status, RB1 mutated and RB1 HD had a significantly higher number of mutated DDR genes (*p* = 0.04 and *p* = 0.003 respectively) as compared with RB1 WT, and RB1 HD compared with RB1 WT also had a higher number of deep deleted DDR genes (p < 0.001) but not RB1 mutants (*p* = 0.064). RB1 HD had a significantly higher number of DDR deleted genes than RB1 mutants (*p* = 0.002) but not of DDR mutated genes (*p* = 0.184).

Finally, according to the TP53 status, for both the TP53 truncated and the TP53 missense mutations, only the number of DDR mutated genes were significantly higher in these mutants when compared with TP53 WT (*p* < 0.001 for both comparisons). However, there was no significant difference between the number of mutated DDR genes in TP53 truncated vs TP53 missense mutants (*p* = 0.136).

Given that the number of alterations in DDR genes have been associated with a higher TMB and a higher number of copy number alterations in MIBCs [20], we checked the potential correlation among the number of DDR mutations and deep deletions with the TMB and number of SNV neoantigens. The TILs fraction was also explored in the estimation of these correlations. The results of these bivariate correlations (Spearman rho) are shown in Table 4.

**Table 4:** Spearman bivariate correlation coefficients of DDR gene mutations, DDR deep deletions, predicted SNV neoantigens, Non-silent muts/Mb (TMB) and TILs fraction

	No. DDR mutations	No. DDR Deep deletions	Predicted SNV neoantigens	TMB	TILs fraction
**No. DDR mutations**	1.00	0.09	0.77**	0.80**	0.26**
**No. DDR Deep deletions**	0.09	1.00	0.17**	0.19**	−0.04
**Predicted SNV neoantigens**	0.77**	0.17**	1.00	0.95**	0.26**
**TMB**	0.80**	0.19**	0.95**	1.00	0.29**
**TILs fraction**	0.26**	−0.04	0.26**	0.29**	1.00

**P value < 0.01

As expected, in this dataset the number of DDR genes deleteriously mutated was strongly correlated with the number of predicted SNV neoantigens (rho = 0.77) and with the TMB (rho = 0.80). A weak correlation was found between the DDR genes affected by deep deletions and SNV neoantigens (rho = 0.17) and with the TMB (rho = 0.19). However, there was no significant correlation between the number of DDR mutated genes and the number of DDR genes affected by deep deletions (rho = 0.09).

The correlation between the TILs fraction and the number of DDR mutations (rho = 0.26), predicted SNV neoantigens (rho = 0.26) and TMB (rho = 0.29) was at best modest although significant. Of note, there was no significant correlation between the TILs fraction and the number of DDR genes affected by deep deletions (rho = − 0.04). This observation is consistent with the data reported in this study where RB1 HDs are not associated with a significant increase in immunological effectors (particularly cytotoxic lymphocytes and NK cells) in the tumor microenvironment as compared with RB1 WT. Furthermore, the increased enrichment of RB1 HD in the TP53 missense mutants might help understand their relative lack of immunological effectors as compared with the TP53 truncated mutants previously commented. Although RB1 was not included as a DDR gen in the data used here from Knijnenburg et al. [21], recent evidence from Cook et al. [22] and Velez-Cruz et al. [23] demonstrate the direct involvement of RB1 in DNA repair by non-homologous end-joining, and in homologous recombination, respectively. Thus, the reports by [22, 23] suggest that RB1 is a bona fide DDR gene.

Next, in order to gain some insight into the relative clinical relevance of the number of DDR mutations, DDR deep deletions, TMB, and the different cell populations and the signatures studied above in the TCGA MIBC dataset along with clinical data available, we explored the prognostic value of these covariates. We used an initial Cox regression model in which the following covariates were included: age (categorical, less than 60 years vs older), stage (categorical, stage II vs stage III and IV), sex, the number of mutated DDR genes (as a continuous covariate) and the TMB (continuous covariate). The number of DDR deep deleted genes was not associated with overall survival in a univariate Cox regression model in the TCGA dataset (data not shown) and hence it was not used for the model. As shown in Table 5, only the age, stage and the TMB were found significant (*p* < 0.05). As expected, older patients (> 60 years old) and a higher stage (III and IV) have a significantly worse overall survival whereas a higher TMB correlated with better survival. Then in a stepwise approach, we have taken the significant covariates (p < 0.05) of this model (age, stage and TMB) and have added 2 cell populations that are not closely related: Tcells and fibroblasts. In addition to age, tumor stage and TMB, Tcells came out as significant (*p* = 0.002, HR = 0.78, 95% CI = 0.66 to 0.91), see Table 6. Then, we removed the non-significant fibroblasts population from the model and tried on a one by one basis the rest of cell populations considered in this work along the 4 covariates identified as significant in the previous model (age, stage, TMB and T cells). None of the 8 remaining cell populations came out as significant, neither the T cell inflamed signature (*p* > 0.05 for the 9 signatures) whereas the core 4 covariates (age, stage, TMB and Tcells) were significant (*p* < 0.05) in each one of the models fitted. The cytolysis signature was dropped from all the survival analysis as it correlated strongly with the T cell inflamed signature (Spearman rho = 0.92).

**Table 5:** Initial Multivariate Cox regression in the TCGA dataset intersecting with data from Knijnenburg et al. [21] (*n* = 399)

	Hazard Ratio	95% CI	***P*** value
**Age** (< 60 vs > 60) **	1.81	1.21–2.70	0.004
**Stage** (II vs III and IV) **	2.14	1.47–2.12	< 0.001
TMB**	0.93	0.89–0.98	0.007
**No. DDR genes mutated**	1.02	0.91–1.13	0.77
**Sex**	1.07	0.76–1.49	0.71

*P* value < 0.05 * < 0.01 **.

**Table 6:** Multivariate Cox regression in the TCGA dataset intersecting with data from Knijnenburg et al. [21] (*n* = 399)

	Hazard Ratio	95% CI	P value
**Age** (< 60 vs > 60) **	1.85	1.24–2.77	**0.003**
**Stage** (II vs III and IV) **	1.90	1.28–2.83	**0.001**
T cells **	0.78	0.66–0.91	**0.002**
TMB **	0.94	0.91–0.97	**< 0.001**
**Fibroblasts**	1.18	0.98–1.42	0.076

*P* value < 0.05 * < 0.01 **.

Of note, the number of infiltrating T cells (CD3+), as reflected by the T cell score, is associated with a better survival as it has been previously reported [24]. In addition, there were not statistically significant differences in this TCGA MIBC dataset in the overall survival according to the RB1 and TP53 mutational status, separately or in combination (logrank test *p* value = 0.998).

### DNA somatic signatures and other recurrent mutations in samples with concurrent genomic alterations in RB1 and TP53 as compared with DWT

**Fig. 1:**
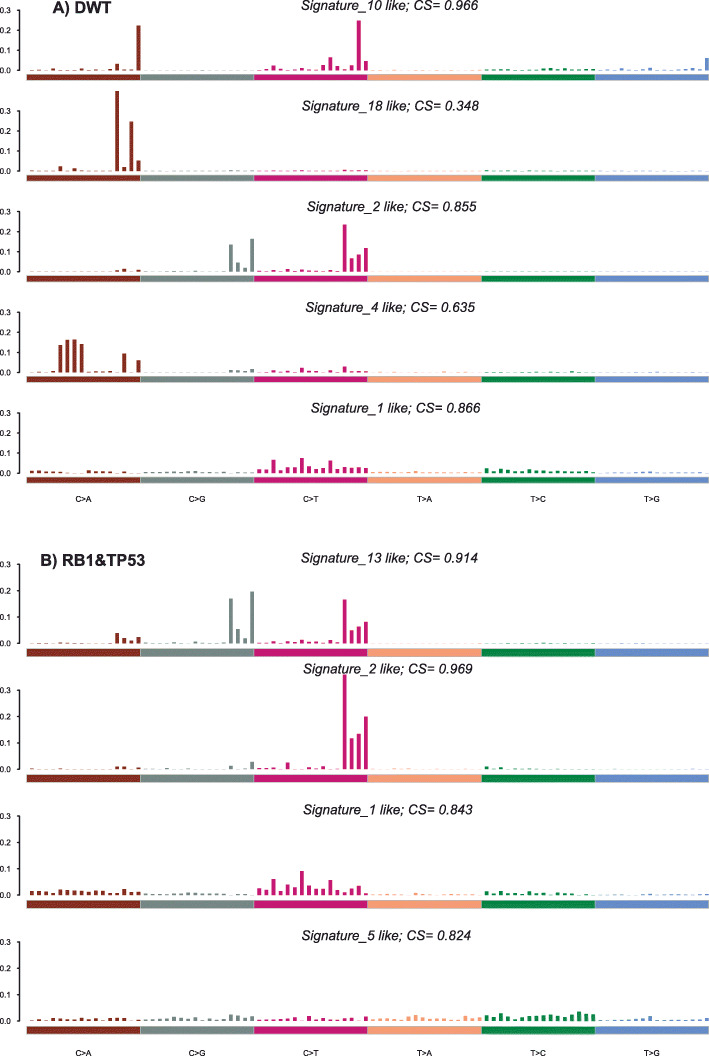
DNA somatic signatures obtained from full exome data in **a** DWT and **b** RB1&TP53. CS refers to the value of Cosine-Similarity

Using the complete exome data from both the double mutants (RB1 and TP53) and the DWT, we extracted the DNA somatic signatures [25] from the trinucleotide SNVs matrices for both groups of samples separately (Fig. 1). In the DWT samples 4 signatures were identified with cosine similarity greater than 0.6: signature 1 (spontaneous deamination of 5-methylcytosine), signature 2 (APOBEC Cytidine Deaminase (C > T)), signature 4 (exposure to tobacco (smoking) mutagens) and signature 10 (defects in polymerase POLE). In the samples with concurrent genomic alterations in RB1 and TP53 4 signatures were also identified: in common with DWT signatures 1 and 2; additionally signature 5 (etiology unknown) and signature 13 (APOBEC Cytidine Deaminase (C > G)). There was a non-significant trend toward a greater number of APOBEC enriched samples in the double mutants (60 out of 69) than in the DWT (147 out of 185), *p* = 0.21, OR = 1.72, 95% CI = 0.76–4.30, Fisher’s exact test. This difference may contribute at least in part to the higher TMB observed in the double RB1 and TP53 altered samples as compared with the DWT. Signature 10 related to defects in polymerase POLE, that is a cause of a hypermutator phenotype and present in DWT, it was only observed in 1 sample of this group, which contained a high mutational load and a POLE mutation (P286R) in the exonuclease domain (predicted deleterious), so it did not contribute much to the overall picture of these samples.

**Fig. 2:**
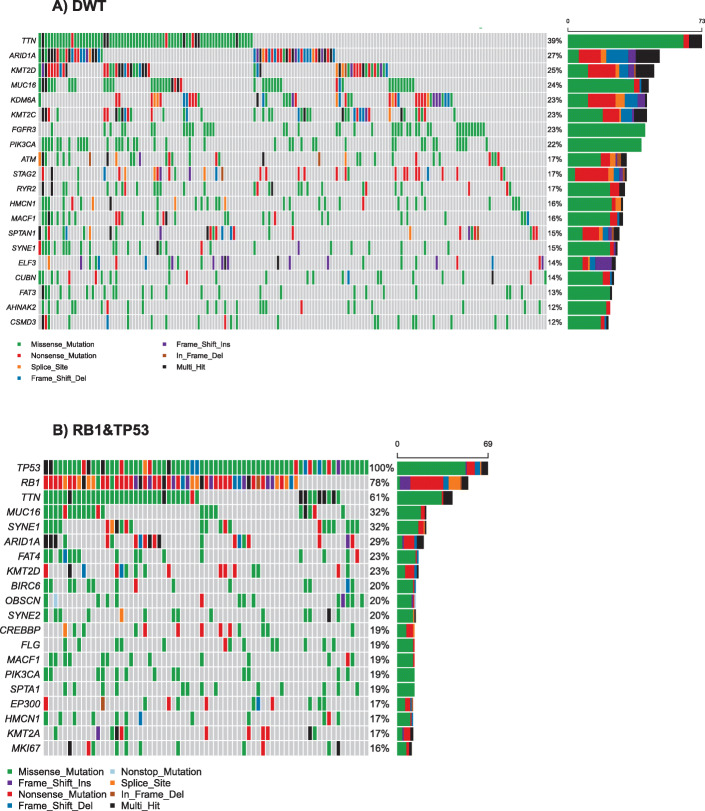
Most recurrent mutations obtained from full exome data in **a** DWT and **b** RB1&TP53, please note that the figure does not include the RB1 HD present in several of the samples but not plotted. Hence, all samples shown in B) are co-altered and have RB1 alterations

The most recurrent mutations present in these two groups of samples (DWT and RB1&TP53) are depicted in Fig. 2. For clarity, this figure does not include copy number alterations. By comparing the overall frequency of the exome mutations detected in these 2 groups of tumors (in at least 5 samples), 2 genes, in addition to RB1 and TP53, were significantly mutated between the double mutants and the DWT: FGFR3 present in 42 samples of the DWT and in none of the double mutants (adjusted *P* value = 0.00063), and RNF17 present in 10 samples of the RB1 and TP53 concurrently mutated samples and just in 1 in the DWT (adjusted P value = 0.0093). When we included, in addition to mutations in FGFR3, amplifications and fusions in this gene, activating genomic events were not found in double RB1 and TP53 concurrently altered samples (0 samples with amplifications or fusions).

### On the relationship of RB1 and TP53 genomic alterations with the response to treatment with immunity checkpoint inhibitors

#### Generation and validation of the tumor microenvironment metascore (TMM) in MIBC and its prognostic value in other tumor types from the TCGA repository

In order to validate some of the observations reported above in the MIBC TCGA dataset of cystectomy specimens, we explored the independent data from the publically available IMvigor 210 study [15], a dataset with 348 metastatic urothelial bladder cancer patients, mostly resistant to previous cisplatin based chemotherapy (78.2%), and all treated with atezolizumab, a PD-L1 inhibitor. From a biopsy of bladder (56%) or a metastatic lesion (42, 2% NA) in each patient, transcriptomic data for all patients, and genomic, clinical and response data for a subset of these patients were available.

First, we determined the most relevant predictive factors influencing the survival of these homogenously treated metastatic patients by building a multivariate Cox regression model with the overall survival data. We obtained the scores for the 11 cell populations from the transcriptomic data as described in the Methods section. Taking the 11 scores as continuous variables along with the TMB as potential predictive molecular factors, a Cox regression model with backward selection (Wald method) of covariates was fitted. The model selected the following statistically significant covariates: CD8Tcells (HR = 0.85, 95% CI = 0.72–1.00, *p* = 0.048), monocytic lineage (HR = 1.47, 95% CI = 1.10–1.97, *p* = 0.01), endothelial cells (HR = 0.75, 95% CI = 0.61–0.92, *p* = 0.006), fibroblasts (HR = 1.26, 95% CI = 1.02–1.54, *p* = 0.03), the T cell inflamed signature (HR = 0.67, 95% CI = 0.48–0.94, *p* = 0.019) and the TMB (HR = 0.97, 95% CI = 0.94–0.99, *p* = 0.002). Likewise, a separate Cox regression with backward selection of clinical covariates was fitted. The clinical covariates selected were: the ECOG performance status (HR = 2.11, 95% CI = 1.55–2.88, *p* < 0.001), previous platinum treatment (HR = 1.49, 95% CI = 1.02–2.17, *p* = 0.038) and the immune cells PD-L1 immunohistochemistry staining (HR = 0.59, 95% CI = 0.40–0.86, *p* = 0.006). The latter refers to the contrast comparing IC0 vs IC2+. Finally, we pulled together the significant covariates from the 2 Cox models to build in a similar way the final model reflecting both clinical and molecular covariates in a single model. The results of the final Cox model with backward selection (Wald method) of the statistically significant covariates selected above (molecular and clinical) were as follows: ECOG performance status (HR = 2.34, 95% CI = 1.67–3.27, *p* < 0.001), CD8Tcells (HR = 0.85, 95% CI = 0.72–0.99, *p* = 0.041), monocytic lineage (HR = 1.44, 95% CI = 1.09–1.91, *p* = 0.011), endothelial cells (HR = 0.79, 95% CI = 0.64–0.96, *p* = 0.021), fibroblasts (HR = 1.28, 95% CI = 1.04–1.58, p = 0.021), T cell inflamed signature (HR = 0.61, 95% CI = 0.46–0.81, *p* = 0.001) and the TMB (HR = 0.96, 95% CI = 0.93–0.98, p < 0.001). The immune cells PD-L1 staining was not selected in the final model but it was borderline significant in the contrast comparing IC0 vs IC2+ (*p* = 0.054), indicating its potential clinical relevance for those patients with the highest percentage of stained immune cells (IC2+) as compared with IC0.

Given the fact that from the transcriptomic data, several different cell populations and the T cell inflamed signature contributed to the final Cox model commented above in this dataset of metastatic patients treated with atezolizumab, we attempted to create a simplified single measurement reflecting the most appropriate combination of cell populations scores (and the T cell inflamed signature) predictive of a better overall survival in these patients. The goal was to achieve a summarization of the most relevant (and favorable) composition of the cell populations and signaling characteristics (as inferred from the T cell inflamed signature) in the tumor microenvironment of those patients having a longer survival with immunotherapy. For this purpose, we used a highly sensitive statistical method based in the Cox model, that tries to shrink to 0 the coefficients of the covariates that contribute the least to the final model, by means of the LASSO penalty [26]. Using this methodology we built and tested a tumor microenvironment metascore comprising the selected combination of cell populations and signature scores that summarizes the best conditions to favor the longest survival after immunotherapy. We hypothesized that this metascore will likely capture some of the known features associated with the response to immunotherapy.

**Fig. 3:**
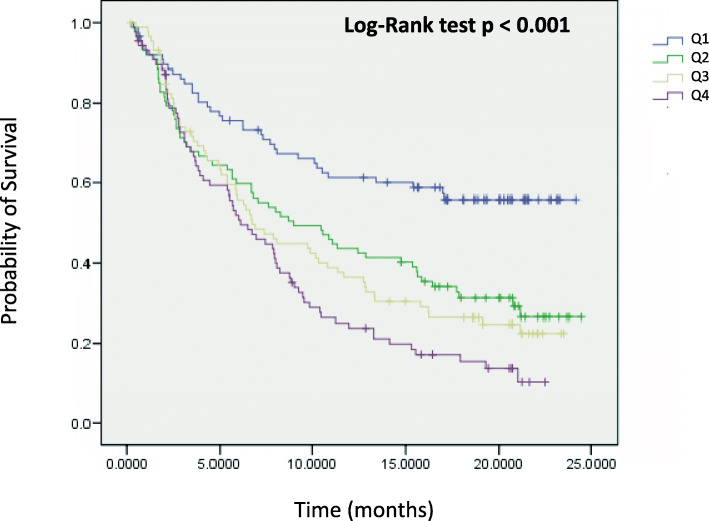
Kaplan-Meier curves of the IMVigor 210 study, using the Tumor Microenvironment Metascore (TMM) as a continuous variable split by quartiles

The tumor microenvironment metascore (TMM) model was validated by 10-fold cross-validation, and out of the 11 scores entered at the minimum lambda parameter, 6 were chosen: CD8Tcells, cytotoxic lymphocytes, NK cells, and the T cell inflamed signature had negative coefficients (higher scores related to a longer survival); and monocytic lineage and fibroblasts had positive coefficients (higher scores related to a worse survival). A higher number (i.e. a higher score) of immunity effectors (CD8Tcells, cytotoxic lymphocytes, NK cells and the T cell inflamed signature) is associated with a better prognosis. Whereas a higher number (i.e. a higher score) of monocytes and fibroblasts is associated with a poorer prognosis. Then the novel tumor microenvironment metascore (TMM) was applied to the 348 patients of this dataset and first used in a Kaplan-Meier curve stratified by quartiles of the continuous metascore (Fig. 3). As shown in Fig. 3 a higher TMM was associated with a statistically significant worse survival (*p* < 0.001, log rank test). Then as a continuous variable in a new multivariate Cox model along with the previously identified significant covariates, i.e. ECOG performance status and TMB (excluding the cell populations and signature scores as the new metascore tested is a linear combination of some of them), and also including the borderline significant immune cell PD-L1 immunohistochemistry. The results are shown in Table 7. Only the ECOG performance status, the TMB and the tumor microenvironment metascore were statistically significant independent prognostic factors (all at *p* < 0.001).

**Table 7:** Cox regression model testing the tumor microenvironment metascore as a continuous variable in the IMvigor 210 study

	Hazard Ratio	95% CI	***P***-value
**ECOG (0 vs 1 or 2)**	2.42	1.73–3.38	< 0.001 ***
**TMB**	0.96	0.94–0.98	< 0.001 ***
IC PD-L1			0.522
IC0 vs IC1	1.04	0.72–1.51	0.843
IC0 vs IC2+	0.84	0.55–1.28	0.416
**Metascore**	4.11	1.97–8.59	< 0.001 ***

^***^ Statistically significant at *p*-value < 0.001

**Fig. 4:**
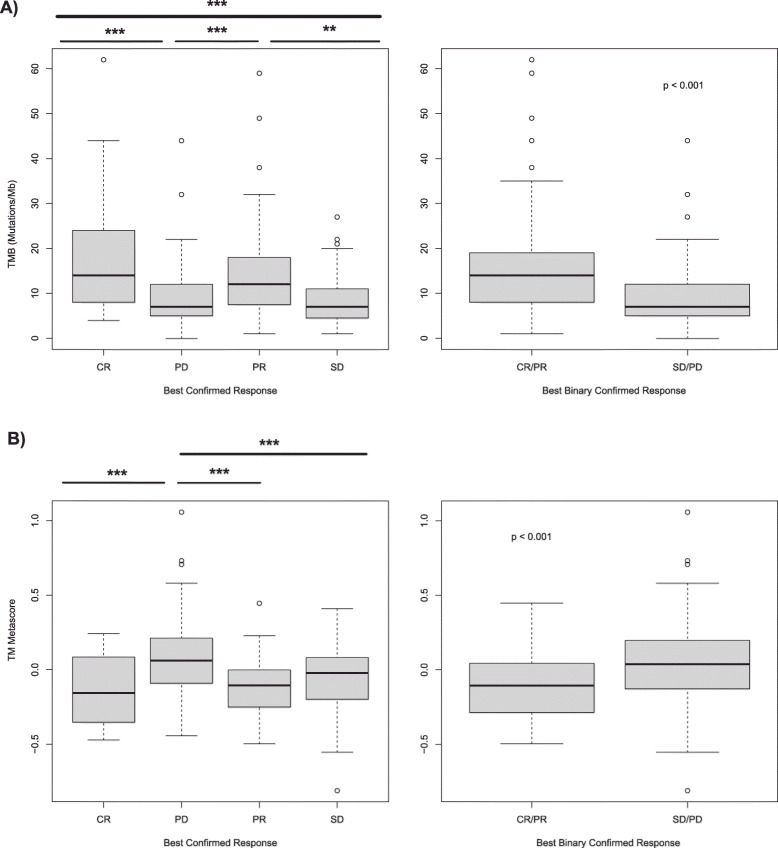
Correlation between the clinical response to atezolizumab in the IMVigor 210 study according to RECIST v1.1 criteria and **a** the Tumor Mutational Burden (TMB) and **b** the TMM. CR: Complete Response; PR: Partial Response; SD: Stable Disease; PD: Progressive disease. ** *P* value < 0.01; *** *P* value < 0.001, Wilcoxon rank sum test, two-sided

Next, we confirmed that the TMM and the TMB were significantly associated with the response to treatment in the IMvigor 210 study (*p* < 0.001 for both, Kruskal-Wallis test) (Fig. 4). There was a modest although significant negative correlation between the two predictors (Spearman rho = − 0.247, *p* < 0.001). Somehow the TMM seems to capture the best favorable conditions of the responding tumor microenvironment as influenced by the cell populations present in it and their signaling, and also as these cell populations are modeled in part by changes in the TMB. These characteristics of the TMM may make it suitable for clinical use in MIBC urothelial cancer along with the TMB although that will require further clinical testing.

**Fig. 5:**
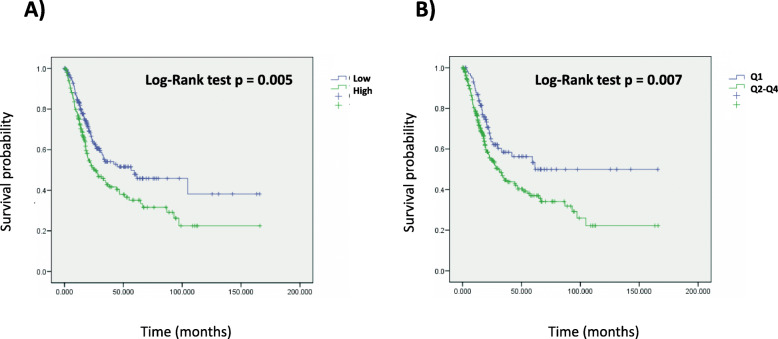
Kaplan Meier curves using the TMM as a continuous variable split by A) the median B) the first quartile in the urothelial bladder cancer TCGA dataset

We wondered whether the TMM has prognostic value in patients that have not received ICIs treatment, as the TMM identifies at least in atezolizumab treated MIBC the most favorable microenvironment conditions correlated with longer survival. For this purpose we estimated the TMM in 20 different tumor types in the TCGA repository for which overall survival and transcriptomic data were available including among them the MIBC TCGA dataset. The median value of the TMM was used to separate patients with high TMM vs those with low TMM. As shown in Fig. 5, in the MIBC TCGA dataset, patients with lower TMM have a significantly better overall survival than those with a higher TMM (*p* = 0.005, log rank test). The TMM as a continuous variable preserved its statistical significance in a Cox model including tumor stage as a categorical covariate (HR = 2.36, 95% CI = 1.30–4.31, *p* = 0.005 for the TMM as a continuous covariate, and HR = 2.13, 95% CI = 1.47–3.08, *p* = 6.62e-05 for the categorical tumor stage). The same was true for the TMM separation using the median value (HR = 1.47, 95% CI = 1.09–1.98, *p* = 0.012 for the categorical TMM split by the median; and HR = 2.14, 95% CI = 1.48–3.11, *p* = 5.59e-05 for the categorical stage) and also using the first TMM quartile (HR = 1.57, 95% CI = 1.09–2.25, *p* = 0.014 for the TMM first quartile split; and HR = 2.15, 95% CI = 1.49–3.12, *p* = 5.07e-05 for the categorical stage covariate).

In the Additional file 2: Figure S1 the Kaplan-Meier curves of the remaining 19 tumors evaluated from the TCGA repository are shown, using the median TMM as a cutoff to separate patients with higher vs lower TMM. In selected cases, where the separation achieved by the median metascore value was not significant but certain separation of the curves was appreciated, the first quartile of the metascore was also shown, as this first quartile provided a better separation of the curves for most of these cases. Significant log-rank tests for using the TMM median separation were seen in 5 tumor types: BRCA (*p* = 0.005), UCEC (*p* = 0.003), LIHC (*p* < 0.001), SKCM (p < 0.001) and THCA (*p* = 0.012); and using the TMM Q1 in 2 tumor types: HNSC (*p* = 0.024) and LUSC (*p* = 0.020). Two other tumor types that showed noticeable separation of the Kaplan-Meier curves with the TMM Q1 curves but no significant log-rank tests were CESC (*p* = 0.074) and STAD (*p* = 0.094). All the tumors mentioned above are tumors in which there are approved indications for ICIs treatment by the drug regulatory agencies with the exception of THCA. The tumors evaluated that did not have neither a significant log-rank test nor a good separation of the Kaplan Meier curves were ACC, COADREAD, ESCA, GBMLGG, KIRC, LUAD, OVCA, PAAD, PRAD, SARC (*p* > 0.05 for all of them). Several of these ones, like GBMLGG, PAAD and PRAD do not have approved indications for the use of ICIs (unless they fulfill the agnostic indication of high instability of microsatellites), and others like LUAD and KIRC have established approved indications for treatment with ICIs. Whether the TMM has also predictive value in other tumor types aside from MIBCs remains to be proven although further studies seem warranted.

#### Correlation with clinical response to atezolizumab in the IMvigor 210 study and with the TMB and TMM in MIBCs with RB1 and TP53 genomic alterations

For this analysis we used a subset of patients (n = 274) from the IMvigor210 study for which mutation data along with transcriptomic and response data were available. Previously we have shown that both the TMB and the TMM had correlation with response to treatment as continuous variables. As categorical variables, using the recently FDA approved cutoff of 10 or more muts/Mb for the TMB, and the first quartile for the TMM, we estimated the AUROC (Area Under the Receiving Operating Characteristics Curve) for both categorical markers as predictors of response: 0.66 for TMB (95% CI = 0.59–0.73) and 0.64 for TMM (95% CI = 0.57–0.71). As a reference point to compare with TMB and TMM, we also estimated the AUROC of PD-L1 immunohistochemistry for IC2+ (i.e. taking as positive the staining of ≥5% immune cells in the tumor microenvironment) in this subset of patients. The AUROC for IC2+ PD-L1 as predictor of response was 0.58 (95% CI = 0.50–0.65). There was no significant difference between the three AUCs (*p* > 0.05 after taking 2000 bootstrap samples for all comparisons). However, the AUROCs of TMB10 and TMMQ1 were superior to the AUROC of IC2+ PD-L1. In addition, we could confirm that TMB10 and TMMQ1 (HR = 0.60, 95% CI 0.43–0.83, *p* = 0.002; and HR = 2.14, 95%CI 1.40–3.28, *p* < 0.001, respectively) were also statistically significant independent predictors of overall survival in a multivariate Cox model including the ECOG performance status (HR = 2.39, 95% CI 1.72–3.33, p < 0.001) and IC2+ PD-L1 (HR = 0.75, 95% CI 0.54–1.04, *p* = 0.087) as categorical factors. Considering the two best predictors in terms of both response and overall survival (TMB10 and TMMQ1), it was interesting to note that patients with these two markers positive had the best chance of response (CR or PR) to atezolizumab (51.1%, 23 out of 45 patients); patients with TMB positive only had a 24.2% response rate (16 out of 66) and patients with TMM positive only had a 20% response rate (6 out of 30); however, patients with both markers negative had 11.3% response rate (15 out of 133).

Regarding the mutational status, 10 patients had RB1 only alterations (3.6%), 101 (36.9%) TP53 only alterations, 33 (12%) had RB1 and TP53 concurrent genomic alterations and 130 (47.4%) were DWT. We found 33.3% response rate (RR, i.e. CR + PR) in the patients with concurrent RB1 and TP53 genomic alterations, 22.2% RR in the DWT, 26.5% RR in patients with TP53 only alterations and 30.0% RR in RB1 only patients, although RB1 patients were very few (10 patients) and this particular result must be interpreted with caution. We did not find a significant enrichment of responding patients in patients with concurrent RB1 and TP53 genomic alterations as compared with DWT (*p* = 0.22, OR = 1.74, 95% CI = 0.61–4.69, Fisher’s exact test). Furthermore, we did not find either, a survival difference in the RB1&TP53 as compared with the DWT (logrank test *p*-value = 0.658).

We also studied the correlation of previously commented response predictive factors in these patients. Specifically, we looked into the TMB and the newly developed TMM as continuous variables. The TMB was significantly higher in the patients with RB1 and TP53 concurrent genomic alterations than in the DWT (*p* = 0.015, Wilcoxon rank sum test). The TMM was significantly lower (hence better correlation with response) in the former as regard the latter (*p* = 0.001, Wilcoxon rank sum test). Then we also compared TMB and TMM in patients with TP53 mutations only vs. RB1 and TP53 concurrent mutated patients. TMB was higher in the RB1 and TP53 concurrent mutated than in the TP53 only mutants although not significantly (*p* = 0.343, Wilcoxon rank sum test), but TMM was significantly lower in the double mutants than in the TP53 only mutants (*p* = 0.031, Wilcoxon rank sum test). In addition, as shown in Table 8, using the cutoff recently approved by the FDA of 10 or more mutations/Mb (TMB10), and the first quartile of TMM (TMMQ1) as categorical variables, there were statistically significant differences in the distribution of both TMB10 and TMMQ1 in patients with RB1 and TP53 concurrent genomic alterations than in those with TP53 mutations only, and in those DWT (TMB10 *p* = 0.037, TMMQ1 *p* = 0.048, Chi square).

**Table 8:** Patients TMB10 + and b) TMMQ1 + according to mutational status

**a)**			
	**TMB10 -**	**TMB10 +**	**Total no. patients**
**DWT**	87	43	**130**
**RB1 only**	7	3	**10**
**TP53 only**	55	46	**101**
**RB1 & TP53**	14	19	**33**
**Chi2,** ***p*** **= 0.037**			
**b)**			
	**TMMQ1 +**	**TMMQ1 -**	**Total no. patients**
**DWT**	29	101	**130**
**RB1 only**	4	6	**10**
**TP53 only**	27	74	**101**
**RB1 & TP53**	15	18	**33**
**Chi2,** ***p*** **= 0.048**			

**Fig. 6:**
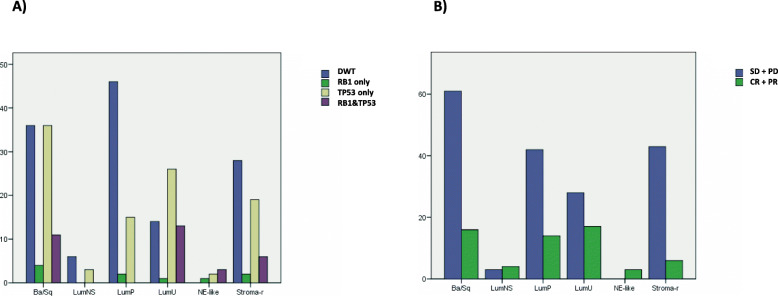
Subset of the IMVigor 210 study (*n* = 274) classified according to the latest consensus molecular subtype classification, separated by **a** Mutational status regarding RB1 and TP53 alterations and **b** binary response to atezolizumab: Responders (CR + PR) and Non-responders (SD + PD)). The y-axis in this figure refers to the number of patients

**Fig. 7:**
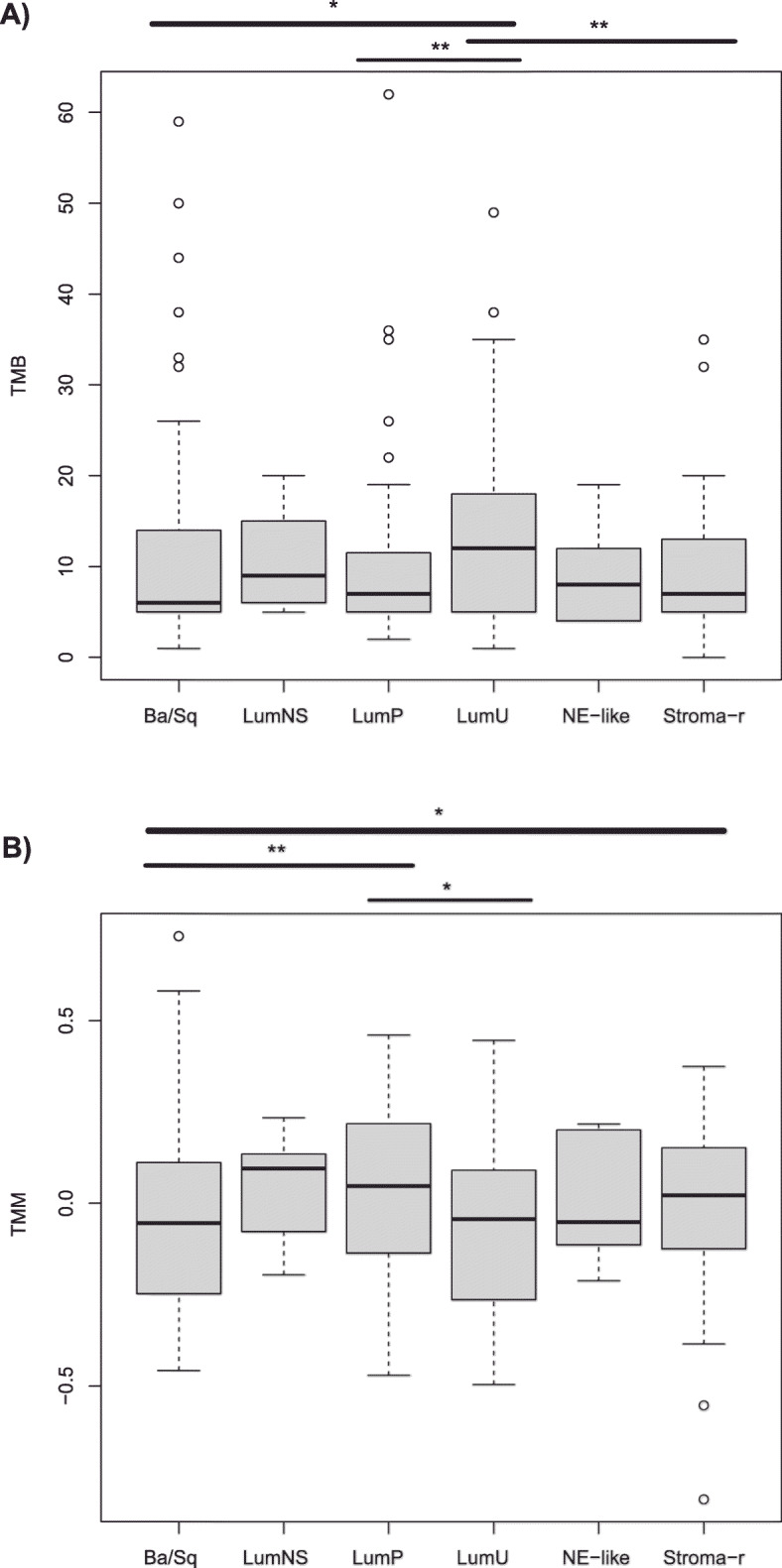
Boxplots showing the differences in **a** TMB and **b** TMM, between the different consensus molecular subtypes. * *P* value < 0.05; ** *P* value < 0.01, Wilcoxon rank sum test, two-sided

To gain further insights, we also applied the latest molecular subtype consensus classification [11] to this subset of the IMvigor210 study (*n* = 274) and could appreciate that there was a correlation between this classification and both the response to treatment (p = 0.001, Chi-square) and the presence of RB1 and TP53 genomic alterations (p < 0.001, Chi-square) (Fig. 6). In order to have a better understanding of the relationship between the response to treatment, the molecular subtypes, and the two predictive factors used above (TMB and TMM), boxplots of the TMB and TMM values as regard the molecular subtypes were plotted (Fig. 7). Seeing Figs. 6 and 7, the 3 molecular subtypes with better response rates were: the neuroendocrine-like (NE-like, 100% RR), luminal non-specified (LumNS, 57.1% RR), and luminal unstable (LumU, 37.8% RR); the worst response rate was in the stroma-rich subtype (Stroma-r, 12.2% RR). Basal squamous (Ba/Sq, 20.8% RR) and luminal papillary (LumP, 25% RR) were intermediate. Some of the best responders, NE-like and LumU had the highest proportion of RB1 and TP53 concurrent mutations (50 and 24% respectively), whereas stroma-rich and Ba/Sq had a lower proportion of double mutants (10.9 and 12.6% respectively) and the lowest median TMB. LumU had the highest median TMB (*p* = 0.02, LumU vs Ba/Sq, Wilcoxon rank sum test). Regarding the TMM LumU, NE-like and Ba/Sq subtypes had the lowest median TMM, and stroma-rich, LumNS and LumP the highest median TMM.

We also wanted to confirm whether there was an absence of FGFR3 mutations in the samples with RB1 and TP53 concurrent genomic alterations in this subset of data as was observed in the TCGA dataset. Indeed, that was the case. No FGFR3 mutations or amplifications were observed in the RB1 and TP53 double mutants in this dataset from the IMvigor210 study, and 36 mutations and 1 amplification in FGFR3 were observed in DWT. There was no data on RNF17 genomic alterations in the IMvigor210 study. However, patients with FGFR3 mutations or amplifications in DWT patients were not enriched in responders as compared with DWT patients lacking FGFR3 mutations (*p* = 0.45, OR = 1.56, 95% CI = 0.54–4.33, Fisher’s exact test).

## Discussion

In this study we focused on the potential influence of concurrent genomic alterations in RB1 and TP53 as modulators of the tumor microenvironment and thus also on the response to ICIs in MIBC. To the best of our knowledge the joint influence of these two tumor suppressor genes, rather than their individual and separate actions, has not been well characterized in this setting. It is interesting to note that bladder urothelial tumors with neuroendocrine features (encompassed in the neuroendocrine-like molecular subtype according to the latest consensus classification) are among the most responsive tumors to ICIs [12]. The presence of the concomitant genomic alterations in these 2 genes occurs in a characteristic way in poorly differentiated neuroendocrine carcinomas [13], but also to a lesser extent in other bladder urothelial carcinomas belonging to different molecular subtypes [11]. It was appealing to think that the joint action of these genes might, to some extent, influence the response to immunotherapy.

First, we demonstrated in the TCGA MIBC dataset that only the tumors with concurrent genomic alterations in RB1 and TP53 had a significant increase in the number of immunity effectors, CD8 cytotoxic lymphocytes and NK cells as compared with the DWT. Tumors with RB1 only or TP53 only genomic alterations did not have such an increase when comparing each one of them with the DWT tumors. The same was true for the T cell inflamed signature, a marker of interferon gamma signaling among immune cells and of response to ICIs [16, 17]. By using other contrasts in our comparisons, it was noted that RB1 HD had a lower modulating impact in the same immunity effectors in the tumor microenvironment. Looking at other TCGA tumor types in which the predominant genomic alteration in RB1 was the HD over other deleterious mutations, like prostate and ovarian cancers, the same phenomenon was observed and no significant differences were found in the amount of immunity effectors between the RB1 HD and the RB1 WT (data not shown).

Second, we confirmed at a FDR < 0.10 the presence in the MIBC TCGA dataset of significantly up- (interferon gamma and alpha) and down- (TGF beta) regulated signaling pathways in the concurrently altered RB1 and TP53 tumors as compared with the DWT in the hallmark collection of signatures. These findings along with the increased presence of immunity effectors and of the T cell inflamed signature discussed earlier have been related to a favorable response to ICIs in previous studies [15–18]. Other pathways that were activated in the concurrently altered RB1 and TP53 tumors were related to an increase in cell proliferation as has been previously reported in bladder urothelial carcinomas [27]. A recent study on clear cell renal carcinoma treated with nivolumab identified the proliferative ability, measured by the mean expression rank value of 10 proliferation related genes, as a response predictor in these patients. Patients with a lower proliferation rate had poorer responses [28]. Furthermore, a recent phase II basket trial of dual blockade with ipilimumab (anti-CTLA4) and nivolumab (anti-PD1) in nonpancreatic neuroendocrine tumors showed an impressive 44% response rate (8 out of 18 patients) in high grade tumors versus 0% (0 out of 14 patients) in low or intermediate grade tumors [29]. Low/intermediate neuroendocrine tumors have by definition lower proliferative ability than high grade tumors which are characterized by a Ki-67 higher than 20% according to the World Health Organization (WHO) G3 classification. Unfortunately, in the latter study no molecular details are given on the high grade tumors.

Third, the well-known predictive factors, like a high TMB and as a consequence an increased number of predicted neoantigens, were found to be statistically significantly associated with the TCGA samples presenting concurrent genomic alterations in RB1 and TP53 as compared with DWT tumors. These observations have been correlated with a higher response rate to ICIs in patients with metastatic bladder urothelial carcinoma [4] and with the acquisition of pT0 after neoadjuvant treatment with pembrolizumab in MIBC [2].

Fourth, our reanalysis identified that the number of DDR mutated and DDR deleted genes were significantly higher in the samples with concurrent genomic alterations in RB1 and TP53 than in the DWT. The presence of DDR mutated genes has been previously correlated with better responses to ICIs in urothelial bladder tumors [19], and also to a higher TMB and a higher number of copy number alterations in these tumors [20]. In fact, we also found a strong correlation between the number of DDR mutated genes and the TMB (Spearman rho = 0.795), but only a weak (but statistically significant) correlation between the TMB and the number of deep deleted DDR genes (Spearman rho = 0.185). The TILs fractions data available as recorded by pathologists in hematoxylin and eosin stained slides from the MIBC TCGA dataset had a modest, but statistically significant, correlation with DDR mutated genes (Spearman rho = 0.257) and with the TMB (Spearman rho = 0.289). It was noticeable that the TILs fractions did not correlate with the number of DDR deep deleted genes (Spearman rho = − 0.036), a fact that could help understand the relative lack of immunity effectors detected in our reanalysis in samples with RB1 HD versus RB1 WT, as we make the case of considering RB1 as a DDR gene according to 2 previous reports [22, 23] showing the direct involvement of this gene in canonical non-homologous end-joining (cNHEJ) and in homologous recombination for the repair of DNA double strand breaks. Velez-Cruz et al. [23] demonstrates the involvement of RB1 in recruiting the ATPase BRG1 to the DNA double strands breaks in an E2F1- and ATM-dependent manner. This recruitment facilitates DNA end resection and homologous recombination. Cook et al. [22] show that RB1 through its N-terminal domain binds XRCC5 and XRCC6 participating in cNHEJ, and that this function is independent of its role in the cell cycle. Therefore, the complete loss of RB1 in RB1 HD, not only prevents its role in controlling the G1/S cell cycle checkpoint but also completely abolishes these 2 DNA repair functions and results in genomic instability in tumors presenting this genomic alteration. However, only a fraction of RB1 mutations affect the RB1 N-terminal domain, where the participation of RB1 in cNHEJ would be impaired. Hence, mutations in other distal domains of the RB1 protein may in some cases possibly retain its ability to bind XRCC5 and XRCC6, and the mutated protein may still be able to function properly in cNHEJ, providing a potential hypothetical explanation for the higher TMB and a significantly higher predicted neoantigen load in the RB1 HD than in the RB1 mutants as a whole, but not for the TILs fractions which are rather similar in these two sample populations in spite of their different average TMB and neoantigen load (please see Table 2 in the Results section). These results do not allow us to assume that RB1 HD are a priori less sensitive to ICIs than the other RB1 mutants, as the RB1 HD have a higher (and almost statistically significant) average TMB and a significantly higher neoantigen load than the rest of the RB1 mutants as a whole, even though RB1 HD also have a lower relative infiltration of immunity relevant cell populations and their signaling (cytotoxic lymphocytes, NK cells, B cells, the monocytic lineage, the T cell inflamed signature and the cytolysis signature) than the rest of RB1 mutants when they are both compared separately with RB1 WT samples (please see the first section of the Results). This lack of correlation of the TILs fractions and the number of DDR deep deletions, considering RB1 as a DDR gene, has already been commented above. Further research will be needed to shed more light on these issues.

In summary, in the MIBC TCGA dataset we found compelling evidence regarding the association of samples with concurrent genomic alterations in RB1 and TP53 (mainly as compared with DWT but also in most of the comparisons with samples with alterations in RB1 alone and in TP53 alone) with factors related to favorable response to treatment with ICIs according to the literature as previously mentioned: 1) increased number of immunity effectors (cytotoxic lymphocytes, NK cells and the T cell inflamed signature) in the tumor microenvironment; 2) the presence of a higher interferon gamma and alpha signatures and a lower TGF beta signaling as well as a higher proliferative ability; 3) a higher TMB, higher neoantigen load and higher TILs fractions; and 4) a higher number of deleteriously mutated and deeply deleted DDR genes.

A potential contribution to the increased TMB in the concurrently altered RB1 and TP53 patients is the increased APOBEC enrichment present in these samples. By looking at the somatic DNA signatures found in the double mutants as compared with the DWT, 2 signatures were in common: signature 1 (spontaneous deamination of 5-methylcytosine) and signature 2 (APOBEC cytidine deaminase (C > T)). But the RB1 and TP53 altered samples also had an additional signature 13 (APOBEC cytidine deaminase (C > G)) not found in DWT. Moreover, APOBEC associated DNA somatic signatures 2 and 13 have been previously related to a higher mutational load and a greater likelihood of response to ICIs in bladder cancer [30]. This fact lends further support to a greater likelihood of response in the DMT (as compared with the DWT) as carriers of this pattern of somatic mutations. Lastly, and consistent with the higher APOBEC enrichment, the expression levels of APOBEC3B were also significantly higher (≈1.5 fold, FDR = 0.016) in the double mutants than in the DWT, but not in the RB1 only nor in the TP53 only mutants as compared independently with the DWT in the TCGA dataset (Supplementary Table 1).

Then, with all the data reanalyzed pointing to a more favorable response to ICIs in the DMT, we explored possibly the largest dataset publically available of metastatic MIBCs treated with atezolizumab to see whether these observations held up. First, we identified the clinical and molecular covariates that correlated better with the longer survivors. With the most significant molecular covariates associated with survival, we built up a single metascore (the TMM) encompassing the best combination of scores representing the studied cell populations (CD8Tcells, cytotoxic lymphocytes, NK cells, the monocytic lineage and fibroblasts) and the T cell inflamed signature. The TMM as a continuous variable was shown to be an independent prognostic factor in this dataset along with the TMB and the ECOG performance status. Moreover, TMM showed prognostic value independently of tumor stage in several TCGA datasets in addition to the MIBC BLCA dataset: SKCM, BRCA, LIHC, UCEC and to a lesser extent HNSC and LUSC, but not in others (like KIRC and LUAD). It is interesting to note that all these mentioned tumor types have established clinical indications for ICIs treatment. As expected, there was also a significant correlation between the response to treatment and the TMM as well as the TMB. We also confirmed that the TMM, used as a categorical covariate (first quartile split, TMMQ1) was also significantly correlated with the response to treatment as well as the recently FDA approved cutoff of 10 or more mutations/Mb (TMB10). Both biomarkers performed similarly in predicting the response to treatment showing non-significantly different AUROCs (0.64 for TMM and 0.66 for TMB). Thus, further research is warranted to explore this novel metascore as a predictor of ICIs response in selected tumor types.

Our results are in full agreement with Cristescu et al. [31], where they show that the TMB and the T cell inflamed signature are predictive biomarkers for pembrolizumab monotherapy effectiveness in several tumor types. These authors showed that patients with both a high TMB and a high value of the T cell inflamed signature had the best RR (37 to 57% depending on tumor type). In our work TMM and TMB are the most important predictors studied, and as categorical variables (TMB10 and TMMQ1), they predict a 51% RR when both biomarkers are favorable in the IMVigor 210 study. However, one biomarker alone (either TMB10 or TMMQ1) is a modest predictor of response (24% RR for TMB10 and 20% RR for TMMQ1).

In one of the largest analysis to date in patients with advanced cancer and multiple tumor types (1662 patients treated with ICIs and 5371 non-ICI treated patients), patients with the highest somatic TMB were associated with better overall survival for most tumor types (including bladder cancer) [32], as we have seen in our reanalysis where TMB (as a continuous and as a discrete variable) had both prognostic and predictive value in MIBC patients treated with atezolizumab. Samstein et al. [32] argue against a universal definition of high TMB, suggesting that the TMB cutpoints related to a better survival benefit are tumor type specific as they are highly variable among tumor types. But recently, FDA approved a TMB ≥ 10 non-synonymous mutations/Mb (as measured with the also FDA approved FoundationOneCDx assay) as an agnostic biomarker of response to the anti-PD1 agent pembrolizumab [33]. Given the clinical relevance of this cutoff, we used it in our reanalysis of the IMVigor 210 dataset, showing its value as a predictor of response in this dataset.

A cautionary note regarding the presence of FGFR3 mutations in DWT MIBCs. It was shown in our reanalysis the complete absence of these FGFR3 mutations in RB1&TP53 co-mutants and its presence in DWT. The presence of FGFR3 mutations or other genomic alterations did not predict a lack of response to atezolizumab in the IMVigor 210 dataset as some researchers had previously hypothesized. Our results are in agreement with the recent study of Necchi et al. [34] where they conclude that patients with MIBCs and FGFR3 genomic alterations should not be excluded from neoadjuvant immunotherapy trials as these authors did not find enough evidence to do so at this time, based on an analysis of a different clinical trial.

We acknowledge two main limitations of our reanalysis: its retrospective nature and the limited number of RB1&TP53 mutants in the IMVigor 210 study (just 33, and only 27 with response data, 9 responders + 18 non-responders). The second limitation is partially overcome by studying response associated biomarkers both in the IMVigor 210 study and in the TCGA dataset (containing 69 patients with RB1&TP53 co-mutants). There was also a good correlation between the clinical response in the different consensus molecular subtypes and the presence of DMT among them.

Therefore, we also have to acknowledge that we could not demonstrate a statistical relationship between RB1&TP53 co-mutants and the response to atezolizumab as compared with the DWT in the IMvigor 210 study, just a modest non-significant increased response rate in DMT. What we did find and could demonstrate is a strong association between these double mutants and several of the known genomic biomarkers of response in the datasets evaluated in this study. Whether there is a link between these associations and a possible response predictive role of these co-mutants in urothelial bladder cancer remains to be proven, and for that to happen a larger number of double mutants with clinical response data will be necessary either to prove or disprove this interesting association.

## Methods

### Datasets

In this work we used The Cancer Genome Atlas (TCGA) dataset of urothelial bladder carcinomas [1] from the Pan-Cancer study, as well as other tumor types. Full mutation (MAF files) and copy number data were downloaded from the cBioportal for Cancer Genomics (https://cbioportal.org). RNA seq v2 (Illumina HiSeq 2000) Level 3 RSEM gene normalized (upper quartile), transcriptomic data was downloaded from the Broad Institute Firebrowser (https://gdac.broadinstitute.org) data from 01 to 28-2016. The IMVigor 210 study dataset was downloaded from Mariathasan et al. [15].

### Cell populations and signature scores. Transcriptome analysis

Highly specific signature genes representing 10 different tissue infiltrating and stromal cell populations were taken from [35]. The genes belonging to 2 other signatures were also used: the 18 genes from a T cell inflamed signature, regarding interferon gamma signaling [16, 17] and the 6 genes from a cytolysis signature [36, 37]. In this reanalysis the genes from these 12 signatures were used to obtain a score for each one of the signatures per sample analyzed in the datasets mentioned earlier (TCGA and IMVigor 210). The way to generate these scores was by applying the single sample gene set enrichment analysis method (ssGSEA) as implemented in the Bioconductor (https://www.bioconductor.org) GSVA R library [38]. This method has been used successfully to quantify tissue infiltrating cell populations showing good correlation with immunohistochemistry and immunofluorescence [35, 39]. ssGSEA is a rank-based method that measures the degree of enrichment of a list of signature genes comparing them with the rest of genes in each RNA seq (or microarray) assay, reflecting the coordinated variation of these lists of genes. It produces a list of almost Gaussians decimals that can be used for statistical analysis. These scores obtained by ssGSEA represent a quantitative measurement of each of the cell populations and signatures considered in this work, and are used for comparisons among the different samples according to their mutational status using non-parametric statistics like the Wilcoxon rank sum test two-sided. Probabilities obtained were adjusted with the Benjamini and Hochberg method to get the false discovery rate (FDR), a FDR < 0.05 was considered significant. Before applying ssGSEA the downloaded normalized TCGA datasets were processed by log2 transformation (RSEM + 1). For the IMVigor 210 dataset, raw count data was normalized within assay by counts per million (CPM) reads per sample, and between assays by upper quartile using the edgeR R library from the Bioconductor project. The voom function from the limma R package (Bioconductor project) was also used along a log2 transformation to stabilize the variance and obtain logCPM. Then the datasets, processing separately the TCGA datasets and the IMVigor 210 data, were ready in turn for applying the ssGSEA method in order to generate the 12 signature scores per sample. For further downstream analysis the obtained scores were standardized (mean = 0, variance = 1).

The 50 Hallmark curated signatures from the MSigDB v.5.1 from the Broad Institute were also used in the TCGA dataset processed as commented above to obtain the 50 ssGSEA scores per sample, and the Wilcoxon rank sum test was also used to compare the scores of the samples according to their mutational status. Likewise *p*-values were adjusted according to the method of Benjamini and Hochberg (FDR). Given the exploratory nature of these pathways comparisons, a different threshold, a FDR < 0.10, was considered significant for this analysis.

### Mutational and copy number analysis

The downloaded RB1 and TP53 missense mutations were assessed using the PolyPhen-2 software [40, 41] to predict the functional effect of the missense mutations in these 2 genes. PolyPhen-2 predicts the effect of single nucleotide variants (SNV) making use of sequence annotations (UniProtKB/Swiss-Prot database), multiple sequence alignments and mapping of the substitution site to protein 3-D structures contained in the protein structure database (PDB). The prediction algorithm of PolyPhen-2 includes 8 sequence-based and 3 structure- based predictive features. The functional effect of an aminoacid replacement is estimated from its individual features by a Naïve Bayes classifier. For the estimation of the functional effects of the missense SNV the HumDiv- trained dataset was used. Missense variants whose posterior probability scores were classified as probably damaging were considered deleterious with high confidence (i.e. ≤ 5% false positive rate, FPR). In this work, we also considered deleterious those mutations whose posterior probability scores were classified as possibly damaging but only those with the highest scores (corresponding to a FPR < 6%, approximately scores > 0.87). It was interpreted in this reanalysis that possibly damaging mutations with lower scores had a milder functional effect (even though that was real) and certainly a lower confidence prediction of deleteriousness. Hence, the PolyPhen-2 score was used here for functional classification of the missense variants with the highest confidence, looking only at the most “dysfunctional” according to the score, taken as an objective measurement.

Non-sense, frameshift (insertion or deletion), splice-site and in-frame (insertion or deletion) mutations were grouped together as deleterious, mostly truncating.

A considerable number of samples had 2 or more mutations of the same gene studied. When 2 or more mutations of one of the genes considered (either RB1 or TP53) were present in the same sample, the functional effect of the most damaging prevailed over non-damaging mutations, and that sample was classified for further analysis as deleterious (example: a sample with a TP53 missense mutation with a low score, say 0.5 in the PolyPhen-2 software, along with a different TP53 second missense mutation with a PolyPhen-2 score of 0.95, will be considered as deleterious for TP53).

Regarding the copy number alterations, we took into consideration as damaging events those samples displaying homozygous deletions (HD) in the genes considered (either RB1 or TP53). Shallow deletions were disregarded. When one sample had simultaneously a HD for one gene and also a mutation in that gene, the sample was labelled as a HD, as the complete loss of a gene by HD would render a mutation ineffective even if it is damaging.

Methylation events were not considered as neither RB1 nor TP53 were found to have evidence of promoter DNA hypermethylation in a recent study regarding a comprehensive molecular characterization of more than 400 TCGA MIBCs [1]. Furthermore, a second independent study characterizing the DNA damage response in the same MIBC TCGA dataset confirmed that there was no evidence of TP53 promoter DNA hypermethylation [21].

Hence, genomic alterations studied included deleterious mutations (as explained above), HD and rearrangements (fusions) that were considered damaging for TP53 and RB1.

To increase statistical power in the comparisons made and gain a greater insight, several contrasts were considered. First, samples were grouped as RB1 altered only (samples with no genomic alterations in TP53), TP53 only (with no RB1 genomic alterations), RB1&TP53 co-altered (simultaneous presence of genomic alterations in both RB1 and TP53) and samples with no genomic alterations in either RB1 or TP53 (double wild type, DWT). Second, tumors with any genomic alterations in RB1 (excluding HD) were labelled as RB1 MT; patients with RB1 HD; and patients with no RB1 genomic alterations (RB1 WT), in the 3 cases in the presence or absence of TP53 genomic alterations. And third, tumors with TP53 missense (MiS) mutations, the rest of TP53 deleterious mutations (mostly truncating mutations) and finally TP53 WT, in the 3 cases samples may or may not have RB1 genomic alterations.

### Tumor mutational burden (TMB), SNV neoantigens and TIL fractions

The tumor mutational burden is the number of non-silent mutations per Megabase of exome DNA, and has been reported in several publications related to the TCGA PanCancer studies [14, 21]. In this report the TMB and the SNV predicted neoantigens have been taken from these studies [14, 21]. The TMB is provided in the IMVigor 210 study from the Foundation One report available along the mutational data.

Tumor Infiltrating Lymphocytes (TILs) fractions were obtained in a subset of patients with MIBCs where digital images were available from Saltz et al. [42]. These authors used a deep learning lymphocyte classification system using a Convolutional Neural Network (CNN) on 5202 digital images of hematoxylin and eosin pathology slides. Each image was divided in 50 × 50 microns squares (called by the authors patches), and the TIL regional fraction was estimated as the number of 50 × 50 squares classified as TIL positive divided by the total number of patches on the tissue image according to the pathologists trained algorithm, expressed as a percentage. Hence this value represents in an intuitive manner the degree of lymphocyte infiltration present in each of the tumors evaluated.

### DNA damage response (DDR) mutated genes and DDR deep deleted genes

Data regarding the number of DDR genes with deleterious mutations and deep deletions was obtained from the study of Knijnenburg et al. [21]. These authors curated a list of 276 genes involved in DDR and study their genomic alterations in the TCGA pancancer dataset across 33 cancer types, including bladder cancer. TP53 belongs to this list of 276 genes but RB1 does not.

### Overall survival and cox regression models. Assessment of response

Overall survival data from the TCGA tumor types was obtained from the Broad Institute Firebrowser as it was the survival data available in a greater number of TCGA tumor types. In the IMVigor 210 study overall survival was also available and provided by the authors [15].

Survival analysis by Kaplan Meier curves was carried out to compare the overall survival of 2 groups of patients, and *p*-values were obtained by log-rank test. Univariate and multivariate Cox regression analysis were performed including the variables indicated in each case, using a Wald test by backward selection of all the variables included in each analysis to select those features with a *p*-value ≤0.05. Hazard ratios and 95% Confidence Intervals were also estimated. SPSS software version 15.0 was used for this purpose.

For the obtention of the Tumor Microenvironment Metascore (TMM) the glmnet R package was used to fit a LASSO regularized Cox regression [26] with 11 out of 12 of the scores generated with the cell populations signatures and with the score of the T cell inflamed signature in the IMVigor 210 study. The score of the cytolysis signature correlated strongly with the T cell inflamed signature (Spearman rho = 0.93) and was dropped from the analysis. The scores used, as mentioned earlier, were standardized before applying this method and those scores coefficients that did not shrink to zero were retained in the model. The model was validated by 10-fold cross-validation. The same score coefficients obtained with this method were later on used without modification as weights of the scores obtained to test the prognostic value of this metascore in 20 different tumor types from the TCGA where overall survival data was available. Transcriptome data was downloaded and processed for each of the tumor types as mentioned in the Datasets methods section. The TMM was tested by Kaplan Meier using the median of the continuous value of the metascore to establish 2 groups of patients. The significance of the separation of the curves was determined by log-rank test. In selected cases the first quartile was used to split the metascore in 2 groups of patients, where a better separation of the curves was expected. In all tumor types tested in which the log-rank test was statistically significant (*p*-value < 0.05), a multivariate Cox regression model was also fitted including as covariates the TMM (as continuous variable) and the stage (categorical) to prove that the metascore retained its significance and that it was independent of stage. In all tumor types fulfilling this criteria (a significant log rank test) that was the case.

Assessment of response to atezolizumab was done by the authors of [15], and it was based on RECIST v1.1. The response assessment was used as provided by these authors in our analysis. Area Under the Receiver Operating Characteristics Curve (AUROC) and its 95% Confidence Interval was estimated with the pROC R library for the categorical predictors of clinical response to atezolizumab used in this analysis (TMMQ1 and TMB10).

### DNA somatic signatures [25] and other recurrent mutations aside from RB1 and TP53

First, the trinucleotide matrix of the 96 possible combinations of mutations and their context was obtained from the exome data using the maftools R package (Bioconductor project) [43]. The matrix was generated separately from the RB1&TP53 samples and from the DWT. In this analysis, the non-synonymous and the synonymous SNV were considered to delineate the mutational patterns. The maftools and the NMF R libraries were used to obtain the signatures present in the samples. The number of signatures (the ranking factor, r) found in the trinucleotide matrix was selected among a range of values based on the first value of r for which the cophenetic correlation coefficient starts decreasing. Then the extracted signatures are compared against 30 validated signatures in the COSMIC database by calculating the cosine similarity.

The unrestricted comparison of all exome mutations aside from RB1 and TP53 between the DWT and the RB1&TP53 samples was performed with the Bioconductor R library maftools [43]. Only those mutations with an adjusted p.value < 0.05 were considered significantly over-represented. A Figure containing the most frequently found mutations in these two groups of samples (DWT and RB1&TP53) was also plotted. In this Figure all exome mutations are considered, not just those that are identified as significant by the MutSig2CV algorithm used by the authors of [1].

### The consensus molecular subtype classification of MIBCs

This classification was applied to the normalized transcriptome data of the IMVigor 210 study by using the R library made available by the authors of [11].

### Other statistical analysis

Non-parametric tests were used for all the statistical analysis referred in this report. Bivariate correlations between quantitative variables were made with the Spearman method. For enrichment of discrete variables Chi-square and Fisher’s exact test were used as appropriate. For comparison of two groups of quantitative variables the Wilcoxon rank sum test, for more than 2, the Kruskal Wallis test was used. All the tests were two-sided. *P* values < 0.05 were considered significant. For most of the analysis and for boxplots the R statistical environment was used. SPSS v15 was also used for bar graphs and for some of the comparisons made.

## Supplementary Information

**Additional file 1: Table S1.** Differential expresión of APOBEC enzymes according to mutational status in the TCGA dataset (expressed as log2 Fold Change, FDR according to Benjamini and Hochberg).

**Additional file 2: Figure S1.**Prognostic value of TMM in different TCGA tumor types

## Data Availability

The datasets analyzed during the current study are available in the TCGA repository that can be accessed through https://www.cbioprotal.org (mutation and copy number data) and through https://gdac.broadinstitute.org/ (transcriptomic and clinical data). The IMVigor 210 study can be obtained from http://research-pub.gene.com/IMvigor210CoreBiologies/IMvigor210CoreBiologies.tar.gz. The coefficients of the tumor microenvironment metascore are available upon reasonable request from the corresponding author.

## Abbreviations

ACC: Adrenocortical Carcinoma
AUROC: Area Under the Receiving Operating Characteristics Curve
BLCA: Bladder Cancer
BRCA: Breast Cancer
Bs/Sq: Basal/Squamous
CESC: **Cervical Squamous Cell Carcinoma and Endocervical Adenocarcinoma**
CI: Confidence Intervals
COADREAD: Colon and rectal adenocarcinomas
DDR: DNA damage Response
DMT: Double Mutants RB1 and TP53
DWT: Double Wild Type
ESCA: Esophageal Carcinoma
FDR: False Discovery Rate
GBMLGG: Glioblastomas and Low Grade Gliomas
HD: Homozygous Deletion
HNSC: Head and Neck Squamous Carcinoma
HR: Hazard Ratio
IC: Immune Cells
ICI: Immunity Checkpoint Inhibitor
KIRC: Kidney Renal Clear Cell Carcinoma
LIHC: Liver Hepatocellular Carcinoma
LUAD: Lung Adenocarcinoma
LumNS: Luminal Non-Specified
LumP: Luminal Papillary
LumU: Luminal Unstable
LUSC: Lung Squamous Carcinoma
MAF: Mutation Annotated Files
MIBC: Muscle Invasive Bladder Cancer
NE-like: Neuroendocrine-like
NK: Natural Killer
OR: Odds Ratio
OVCA: Ovarian Carcinoma
PAAD: Pancreatic Adenocarcinoma
PDB: Protein Structure Database
PRAD: Prostatic Adenocarcinoma
Q1: First Quartile
SARC: Sarcoma
SKCM: Skin Cutaneous Melanoma
SNV: Single Nucleotide Variant
ssGSEA: Single Sample Gene Set Enrichment Analysis
**STAD**: **Stomach Adenocarcinoma**
Stroma-r: Stroma-rich
TCGA: The Cancer Genome Atlas
THCA: Thyroid Carcinoma
TILs: Tumor Infiltrating Lymphocytes
TMB: Tumor Mutational Burden
TMM: Tumor Microenvironment Metascore
UCEC: Uterine Corpus Endometrial Carcinoma
WT: Wild Type

## References

[1] Robertson AG, Kim J, Al-Ahmadie H, Bellmunt J, Guo G, Cherniack AD, Hinoue T, Laird PW, Hoadley KA, Akbani R *et al*: Comprehensive molecular characterization of muscle-invasive bladder Cancer. Cell 2017, 171(3):540–556 e525. 10.1016/j.cell.2017.09.007 PMC5687509 28988769

[2] Necchi A, Anichini A, Raggi D, Briganti A, Massa S, Lucianò R, et al. Pembrolizumab as Neoadjuvant Therapy Before Radical Cystectomy in Patients With Muscle-Invasive Urothelial Bladder Carcinoma (PURE-01): An Open-Label, Single-Arm, Phase II Study. J Clin Oncol. 2018:Jco1801148. 10.1200/JCO.18.01148 30343614

[3] Powles T, Duran I, van der Heijden MS, Loriot Y, Vogelzang NJ, De Giorgi U, Oudard S, Retz MM, Castellano D, Bamias A (2018). Atezolizumab versus chemotherapy in patients with platinum-treated locally advanced or metastatic urothelial carcinoma (IMvigor211): a multicentre, open-label, phase 3 randomised controlled trial. Lancet (London, England).

[4] Rosenberg JE, Hoffman-Censits J, Powles T, van der Heijden MS, Balar AV, Necchi A, Dawson N, O'Donnell PH, Balmanoukian A, Loriot Y (2016). Atezolizumab in patients with locally advanced and metastatic urothelial carcinoma who have progressed following treatment with platinum-based chemotherapy: a single-arm, multicentre, phase 2 trial. Lancet (London, England).

[5] Powles T, Kockx M, Rodriguez-Vida A, Duran I, Crabb SJ, Van Der Heijden MS, Szabados B, Pous AF, Gravis G, Herranz UA (2019). Clinical efficacy and biomarker analysis of neoadjuvant atezolizumab in operable urothelial carcinoma in the ABACUS trial. Nat Med.

[6] Cancer Genome Atlas Research Network. Comprehensive molecular characterization of urothelial bladder carcinoma. Nature. 2014;507(7492):315–22. 10.1038/nature12965 PMC3962515 24476821

[7] Choi W, Porten S, Kim S, Willis D, Plimack ER, Hoffman-Censits J, Roth B, Cheng T, Tran M, Lee IL (2014). Identification of distinct basal and luminal subtypes of muscle-invasive bladder cancer with different sensitivities to frontline chemotherapy. Cancer Cell.

[8] Damrauer JS, Hoadley KA, Chism DD, Fan C, Tiganelli CJ, Wobker SE, Yeh JJ, Milowsky MI, Iyer G, Parker JS (2014). Intrinsic subtypes of high-grade bladder cancer reflect the hallmarks of breast cancer biology. Proc Natl Acad Sci U S A.

[9] Sjodahl G, Lauss M, Lovgren K, Chebil G, Gudjonsson S, Veerla S, Patschan O, Aine M, Ferno M, Ringner M (2012). A molecular taxonomy for urothelial carcinoma. Clin Cancer Res.

[10] Choi W, Ochoa A, McConkey DJ, Aine M, Hoglund M, Kim WY, Real FX, Kiltie AE, Milsom I, Dyrskjot L (2017). Genetic alterations in the molecular subtypes of bladder Cancer: illustration in the Cancer genome atlas dataset. Eur Urol.

[11] Kamoun A, de Reyniès A, Allory Y, Sjödahl G, Robertson AG, Seiler R, Hoadley KA, Groeneveld CS, Al-Ahmadie H, Choi W (2020). A consensus molecular classification of muscle-invasive bladder Cancer. Eur Urol.

[12] Kim J, Kwiatkowski D, McConkey DJ, Meeks JJ, Freeman SS, Bellmunt J, Getz G, Lerner SP (2019). The Cancer genome atlas expression subtypes stratify response to checkpoint inhibition in advanced Urothelial Cancer and identify a subset of patients with high survival probability. Eur Urol.

[13] Rickman DS, Beltran H, Demichelis F, Rubin MA (2017). Biology and evolution of poorly differentiated neuroendocrine tumors. Nat Med.

[14] Thorsson V, Gibbs DL, Brown SD, Wolf D, Bortone DS, Ou Yang TH, Porta-Pardo E, Gao GF, Plaisier CL, Eddy JA *et al*: The immune landscape of Cancer. Immunity 2018, 48(4):812–830 e814. 10.1016/j.immuni.2018.03.023 PMC5982584 29628290

[15] Mariathasan S, Turley SJ, Nickles D, Castiglioni A, Yuen K, Wang Y, Kadel EE, Koeppen H, Astarita JL, Cubas R (2018). TGFbeta attenuates tumour response to PD-L1 blockade by contributing to exclusion of T cells. Nature.

[16] Ayers M, Lunceford J, Nebozhyn M, Murphy E, Loboda A, Kaufman DR, Albright A, Cheng JD, Kang SP, Shankaran V (2017). IFN-γ-related mRNA profile predicts clinical response to PD-1 blockade. J Clin Invest.

[17] Ott PA, Bang YJ, Piha-Paul SA, Razak ARA, Bennouna J, Soria JC, Rugo HS, Cohen RB, O'Neil BH, Mehnert JM (2019). T-cell-inflamed gene-expression profile, programmed death ligand 1 expression, and tumor mutational burden predict efficacy in patients treated with Pembrolizumab across 20 cancers: KEYNOTE-028. J Clin Oncol.

[18] Necchi A, Raggi D, Gallina A, Ross JS, Farè E, Giannatempo P, et al. Impact of molecular subtyping and immune infiltration on pathological response and outcome following Neoadjuvant Pembrolizumab in muscle-invasive bladder Cancer. Eur Urol. 2020;77(6):701–10. 10.1016/j.eururo.2020.02.028 32165065

[19] Teo MY, Seier K, Ostrovnaya I, Regazzi AM, Kania BE, Moran MM, Cipolla CK, Bluth MJ, Chaim J, Al-Ahmadie H (2018). Alterations in DNA damage response and repair genes as potential marker of clinical benefit from PD-1/PD-L1 blockade in advanced Urothelial cancers. J Clin Oncol.

[20] Teo MY, Bambury RM, Zabor EC, Jordan E, Al-Ahmadie H, Boyd ME, Bouvier N, Mullane SA, Cha EK, Roper N (2017). DNA damage response and repair gene alterations are associated with improved survival in patients with platinum-treated advanced Urothelial carcinoma. Clin Cancer Res.

[21] Knijnenburg TA, Wang L, Zimmermann MT, Chambwe N, Gao GF, Cherniack AD, Fan H, Shen H, Way GP, Greene CS *et al*: Genomic and Molecular Landscape of DNA Damage Repair Deficiency across The Cancer Genome Atlas. Cell Rep 2018, 23(1): 239–254.e236. 10.1016/j.celrep.2018.03.076 PMC5961503 29617664

[22] Cook R, Zoumpoulidou G, Luczynski MT, Rieger S, Moquet J, Spanswick VJ, Hartley JA, Rothkamm K, Huang PH, Mittnacht S (2015). Direct involvement of retinoblastoma family proteins in DNA repair by non-homologous end-joining. Cell Rep.

[23] Velez-Cruz R, Manickavinayaham S, Biswas AK, Clary RW, Premkumar T, Cole F, Johnson DG (2016). RB localizes to DNA double-strand breaks and promotes DNA end resection and homologous recombination through the recruitment of BRG1. Genes Dev.

[24] Yu A, Mansure JJ, Solanki S, Siemens DR, Koti M, Dias ABT, Burnier MM, Brimo F, Kassouf W (2018). Presence of lymphocytic infiltrate cytotoxic T lymphocyte CD3+, CD8+, and immunoscore as prognostic marker in patients after radical cystectomy. PLoS One.

[25] Alexandrov LB, Nik-Zainal S, Wedge DC, Aparicio SA, Behjati S, Biankin AV, Bignell GR, Bolli N, Borg A, Borresen-Dale AL (2013). Signatures of mutational processes in human cancer. Nature.

[26] Simon N, Friedman JH, Hastie T, Tibshirani R. Regularization Paths for Cox's Proportional Hazards Model via Coordinate Descent. *2011* 2011, **39**(5):13. 10.18637/jss.v039.i05 PMC4824408 27065756

[27] Goussia AC, Papoudou-Bai A, Charchanti A, Kitsoulis P, Kanavaros P, Kalef-Ezra J, Stefanou D, Agnantis NJ (2018). Alterations of p53 and Rb pathways are associated with high proliferation in bladder Urothelial carcinomas. Anticancer Res.

[28] Zhang T, Pabla S, Lenzo FL, Conroy JM, Nesline MK, Glenn ST, Papanicolau-Sengos A, Burgher B, Giamo V, Andreas J (2020). Proliferative potential and response to nivolumab in clear cell renal cell carcinoma patients. OncoImmunology.

[29] Patel SP, Othus M, Chae YK, Giles FJ, Hansel DE, Singh PP, Fontaine A, Shah MH, Kasi A, Baghdadi TA (2020). A phase II basket trial of dual anti–CTLA-4 and anti–PD-1 blockade in rare Tumors (DART SWOG 1609) in patients with nonpancreatic neuroendocrine Tumors. Clin Cancer Res.

[30] Miao D, Margolis CA, Vokes NI, Liu D, Taylor-Weiner A, Wankowicz SM, Adeegbe D, Keliher D, Schilling B, Tracy A (2018). Genomic correlates of response to immune checkpoint blockade in microsatellite-stable solid tumors. Nat Genet.

[31] Cristescu R, Mogg R, Ayers M, Albright A, Murphy E, Yearley J, Sher X, Liu XQ, Lu H, Nebozhyn M (2018). Pan-tumor genomic biomarkers for PD-1 checkpoint blockade-based immunotherapy. Science (New York, NY).

[32] Samstein RM, Lee C-H, Shoushtari AN, Hellmann MD, Shen R, Janjigian YY, Barron DA, Zehir A, Jordan EJ, Omuro A (2019). Tumor mutational load predicts survival after immunotherapy across multiple cancer types. Nat Genet.

[33] FDA approves pembrolizumab for adults and children with TMB-H solid tumors. https://www.fda.gov/drugs/drug-approvals-and-databases/fda-approves-pembrolizumab-adults-and-childrentmb-h-solid-tumors. Accessed July 2020.

[34] Necchi A, Raggi D, Giannatempo P, Marandino L, Farè E, Gallina A, et al. Can patients with muscle-invasive bladder Cancer and fibroblast growth factor Receptor-3 alterations still be considered for Neoadjuvant Pembrolizumab? A comprehensive assessment from the updated results of the PURE-01 study. Eur Urol. 2020;77(4):439–46. 10.1016/j.euo.2020.04.005 32417369

[35] Becht E, Giraldo NA, Lacroix L, Buttard B, Elarouci N, Petitprez F, Selves J, Laurent-Puig P, Sautès-Fridman C, Fridman WH (2016). Estimating the population abundance of tissue-infiltrating immune and stromal cell populations using gene expression. Genome Biol.

[36] Wei JS, Kuznetsov IB, Zhang S, Song YK, Asgharzadeh S, Sindiri S, Wen X, Patidar R, Najaraj S, Walton A (2018). Clinically relevant cytotoxic immune cell signatures and clonal expansion of T-cell receptors in high-risk MYCN-not-amplified human neuroblastoma. Clin Cancer Res.

[37] Yang RK, Kuznetsov IB, Ranheim EA, Wei JS, Sindiri S, Gryder BE, Gangalapudi V, Song YK, Patel V, Hank JA (2020). Outcome-related signatures identified by whole Transcriptome sequencing of Resectable stage III/IV melanoma evaluated after starting Hu14.18-IL2. Clin Cancer Res.

[38] Hanzelmann S, Castelo R, Guinney J (2013). GSVA: gene set variation analysis for microarray and RNA-seq data. BMC Bioinform.

[39] Şenbabaoğlu Y, Gejman RS, Winer AG, Liu M, Van Allen EM, de Velasco G, Miao D, Ostrovnaya I, Drill E, Luna A (2016). Tumor immune microenvironment characterization in clear cell renal cell carcinoma identifies prognostic and immunotherapeutically relevant messenger RNA signatures. Genome Biol.

[40] Adzhubei I, Jordan DM, Sunyaev SR: Predicting functional effect of human missense mutations using PolyPhen-2. Curr Protoc Hum Genet 2013, Chapter 7: Unit 7 20. 10.1002/0471142905.hg0720s76 PMC4480630 23315928

[41] Adzhubei IA, Schmidt S, Peshkin L, Ramensky VE, Gerasimova A, Bork P, Kondrashov AS, Sunyaev SR (2010). A method and server for predicting damaging missense mutations. Nat Methods.

[42] Saltz J, Gupta R, Hou L, Kurc T, Singh P, Nguyen V, Samaras D, Shroyer KR, Zhao T, Batiste R *et al*: Spatial organization and molecular correlation of tumor-infiltrating lymphocytes using deep learning on pathology images. Cell Rep 2018, 23(1):181–193 e187. 10.1016/j.celrep.2018.03.086 PMC5943714 29617659

[43] Mayakonda A, Lin DC, Assenov Y, Plass C, Koeffler HP (2018). Maftools: efficient and comprehensive analysis of somatic variants in cancer. Genome Res.

