# Supplementary figures and images for: RB1 and TP53 co-mutations correlate strongly with genomic biomarkers of response to immunity checkpoint inhibitors in urothelial bladder cancer
Source: BMC Cancer. 2021 Apr 20;21:432. doi: 10.1186/s12885-021-08078-y (PMC8056512; doi:10.1186/s12885-021-08078-y)

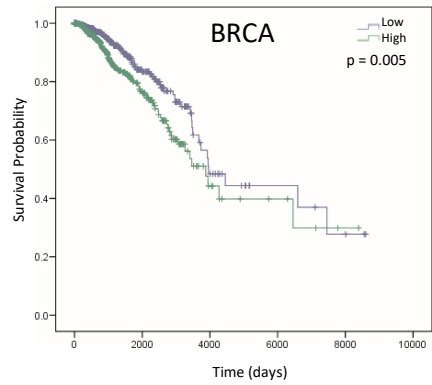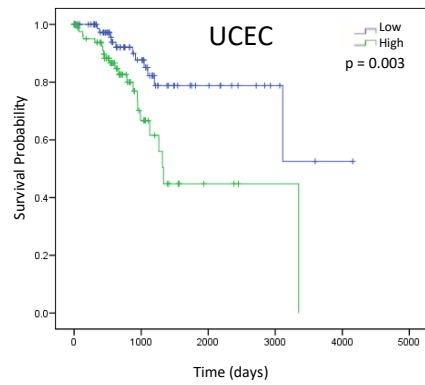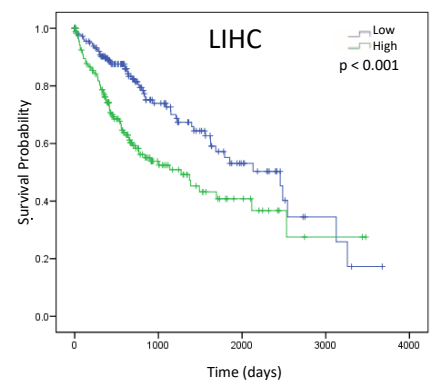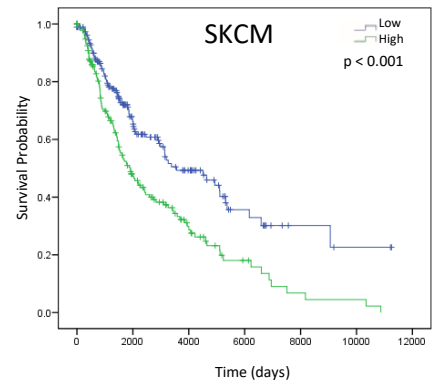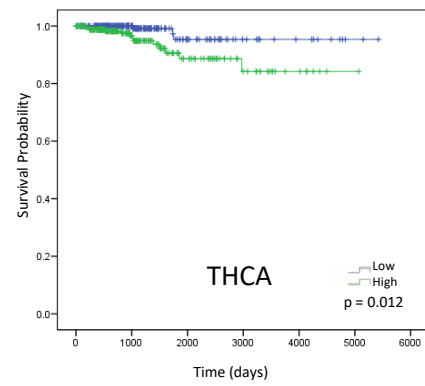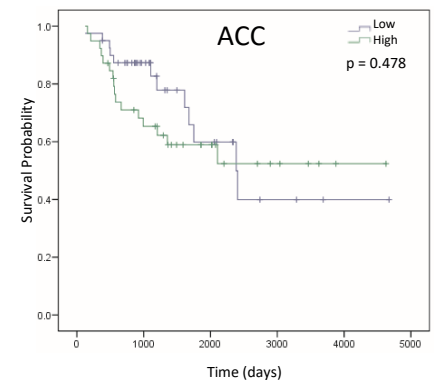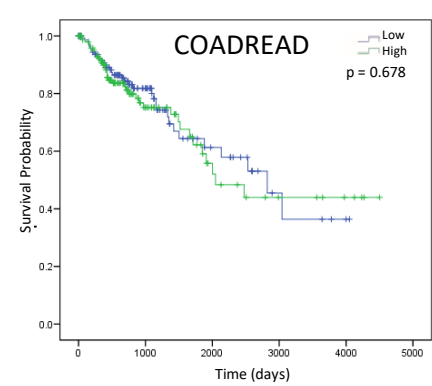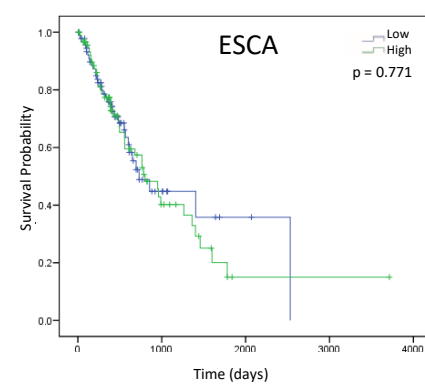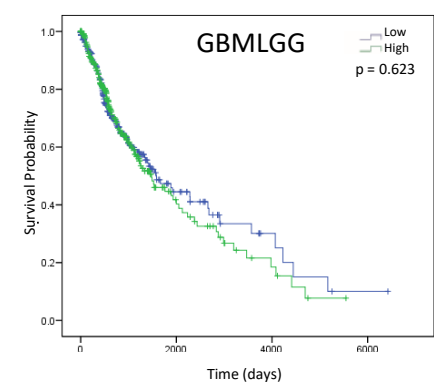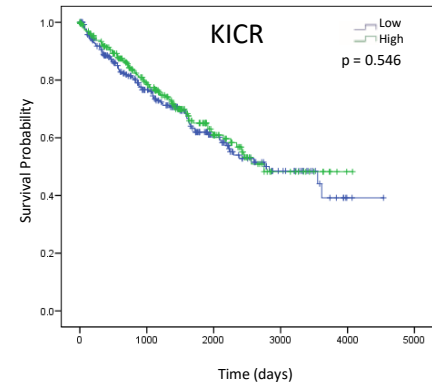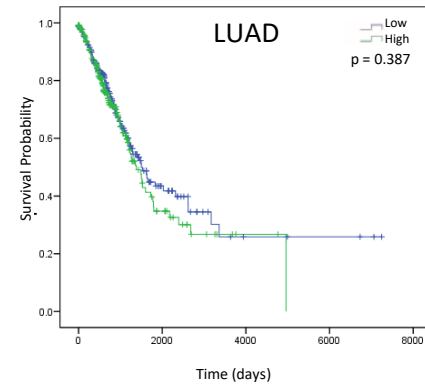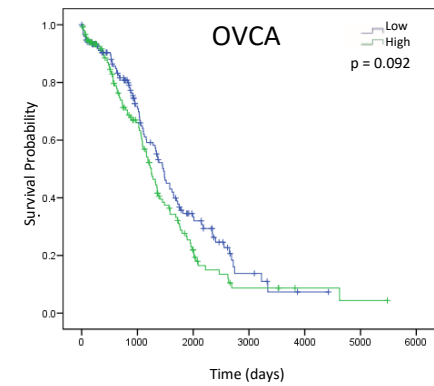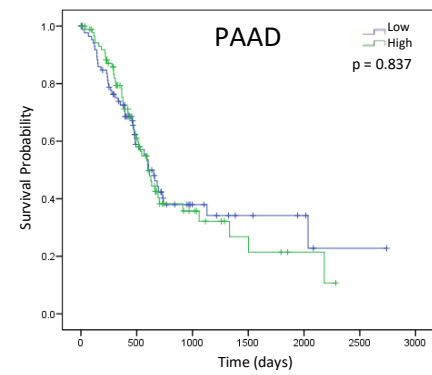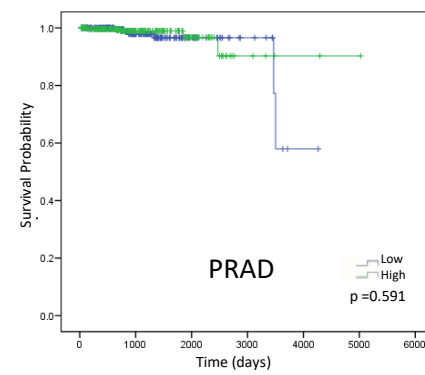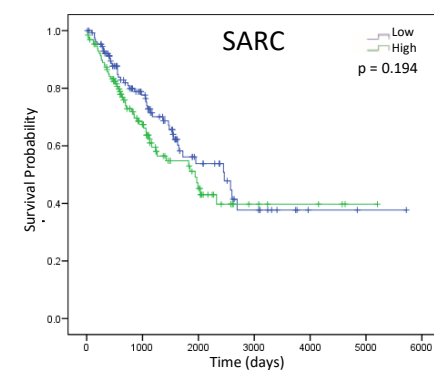

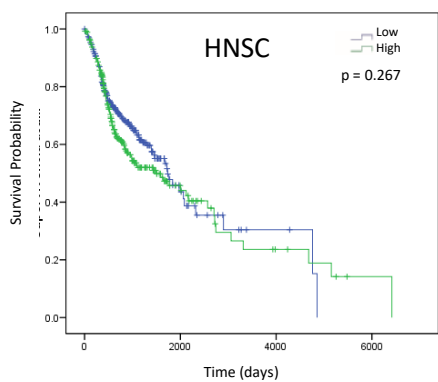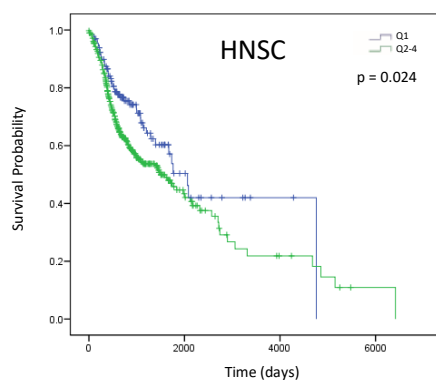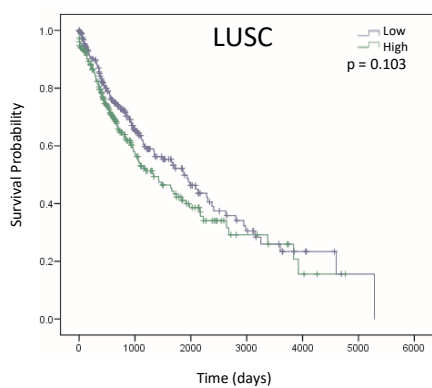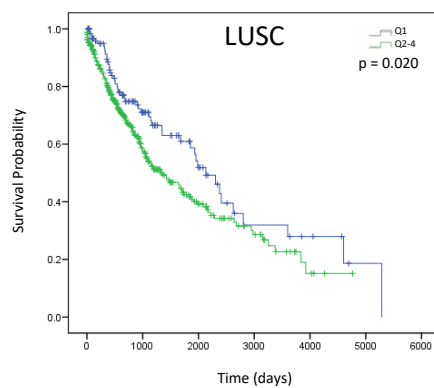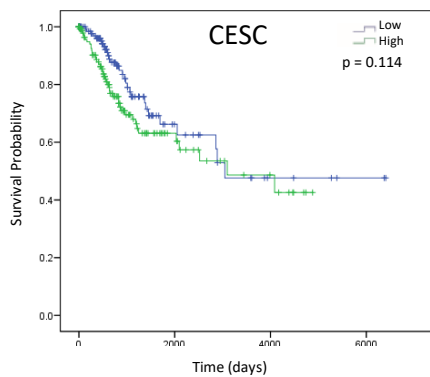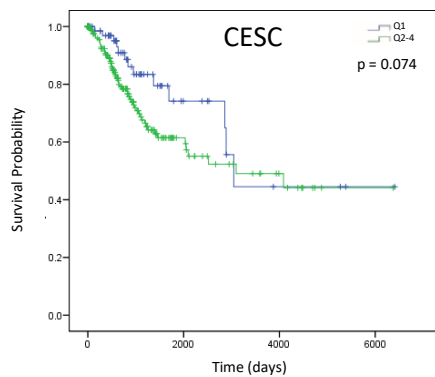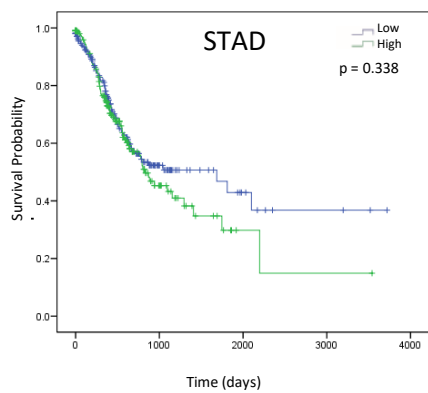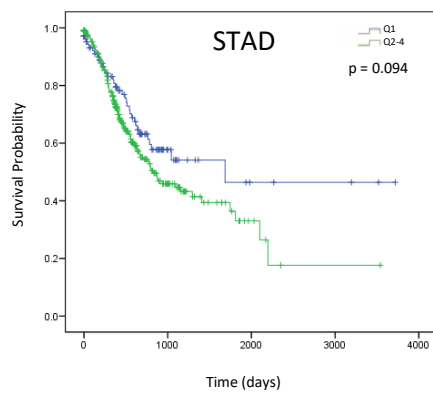

Supplement: Supplementary file 1 — Additional file 1: Table S1. Differential expresión of APOBEC enzymes according to mutational status in the TCGA dataset (expressed as log2 Fold Change, FDR according to Benjamini and Hochberg). [file 12885_2021_8078_MOESM1_ESM.docx]
